# *In Vitro* detection of Chronic Wasting Disease (CWD) prions in semen and reproductive tissues of white tailed deer bucks *(Odocoileus virginianus)*

**DOI:** 10.1371/journal.pone.0226560

**Published:** 2019-12-30

**Authors:** Carlos Kramm, Ruben Gomez-Gutierrez, Claudio Soto, Glenn Telling, Tracy Nichols, Rodrigo Morales

**Affiliations:** 1 Department of Neurology, McGovern Medical School, The University of Texas Health Science Center at Houston, Houston, TX, United States of America; 2 Universidad de Los Andes, Facultad de Medicina, Las Condes, Santiago, Chile; 3 Department of Cell Biology, University of Malaga, Malaga, Spain; 4 Prion Research Center, Colorado State University, Fort Collins, CO, United States of America; 5 Veterinary Services, APHIS, United States Department of Agriculture, Fort Collins, CO, United States of America; 6 CIBQA, Universidad Bernardo OHiggins. Santiago, Chile; INRA Centre de Jouy-en-Josas, FRANCE

## Abstract

Chronic Wasting Disease (CWD) is a prion disease affecting several cervid species. Among them, white-tailed deer (WTD) are of relevance due to their value in farming and game hunting. The exact events involved in CWD transmission in captive and wild animals are still unclear. An unexplored mechanism of CWD spread involves transmissions through germplasm, such as semen. Surprisingly, the presence and load of CWD prions in semen and male sexual tissues from WTD has not been explored. Here, we described the detection of CWD prions in semen and sexual tissues of WTD bucks utilizing the Protein Misfolding Cyclic Amplification (PMCA) technology. Samples were obtained *post-mortem* from farmed pre-clinical, CWD positive WTD bucks possessing polymorphisms at position 96 of the *PRNP* gene. Our results show that overall CWD detection in these samples had a sensitivity of 59.3%, with a specificity of 97.2%. The data indicate that the presence of CWD prions in male sexual organs and fluids is prevalent in late stage, pre-clinical, CWD-infected WTD (80%-100% of the animals depending on the sample type analyzed). Our findings reveal the presence of CWD prions in semen and sexual tissues of prion infected WTD bucks. Future studies will be necessary to determine whether sexual contact and/or artificial inseminations are plausible means of CWD transmission in susceptible animal species.

## Introduction

Chronic Wasting Disease (CWD) is a prion disease affecting cervids including deer, elk, reindeer and moose [1–3]. CWD is unique among prionopathies as it is currently the only transmissible spongiform encephalopathy (TSE) identified in wild animals [3,4]. CWD continues to spread across North America with 26 States within the United States and 3 Canadian provinces having documented CWD in wild and/or captive cervids (https://www.usgs.gov/media/images/distribution-chronic-wasting-disease-north-america-0). CWD-infected animals have been identified in Asia (South Korea, [5,6]) and more recently in Northern Europe [3].

Although the mechanisms of CWD spread are not fully understood, it is thought direct nose to nose contact and indirect contact via environmental contamination play major roles in this process [7,8]. CWD prions are known to be shed into the environment by urine, feces and saliva released from infected animals [9–13]. The progressive accumulation of prions in the environment by shedding, carcasses decomposition and other tissue sources over time, coupled with the environmental persistence and resistance to degradation of this particular infectious agent, make a compelling argument as to the role of the environment contamination in CWD transmission in both natural and captive settings. Nevertheless, other scenarios contributing to CWD transmission have also been proposed. These involve the appearance of sporadic CWD cases (analogous of sporadic Creutzfeldt-Jakob disease (sCJD) in humans), translocation of the infectious agent by scavengers [14,15], and vertical transmission from mother to offspring [16]. Transmission through sexual contact and semen is a logical line of inquiry that, surprisingly, has not been fully explored in CWD research. The first step in this process is to determine if infectious CWD prions are present in the semen and testes of CWD-infected cervids.

One of the main challenges to the detection of infectious prions in samples other than brain and lymph nodes is the presumably low concentrations present on them. Recently, we described an adapted version of the Protein Misfolding Cyclic Amplification (PMCA) technology for the ultrasensitive detection of CWD prions in blood [17]. Our modified parameters allowed us to detect prion infectivity in highly diluted brain materials estimated to be at sub-infectious quantities. Using this CWD-specific PMCA settings, we were able to detect CWD prions in the blood of pre-symptomatic white-tailed deer (WTD) with great sensitivity and specificity [17]. Taking advantage of these technical improvements, we explored the presence of CWD prions in semen and male reproductive tissues of farmed, naturally infected WTD bucks at different stages of the CWD course.

## Materials and methods

### Ethics statements

Deer testes were collected *post mortem* from captive animals depopulated due to the presence of CWD, or CWD-free facilities. Tg1536 mice (overexpressing the white-tailed deer prion protein harboring the 96G polymorphic version, [18]) were used following regulations provided by the Center of Laboratory Animal Medicine and Care (CLAMC) of The University of Texas Health Science Center at Houston.

### Samples

Semen and sexual tissues (testes stroma and epididymides) were collected by USDA personnel from 9 WTD bucks from captive cervids in various locations across the United States. Additional samples were collected from 12 WTD from CWD-free facilities and used as negative controls. CWD-derived samples were obtained from animals at early (n = 4) and late (n = 5) stages of the prion incubation period. This classification was assigned based on *post-mortem* immunohistochemical examination of PrP^Sc^ deposition in the medial retropharyngeal lymph nodes (MRPLN) and brain stem (obex). Specifically, PrP^Sc^ staining present only in the MRPLN was considered early pre-symptomatic, whereas PrP^Sc^ detection in both the MRPLN and obex was considered to be late pre-symptomatic. Animals in this study had prion protein polymorphic variations at codon 96 (96GG, n = 5; 96GS, n = 2; 96SS, n = 2). Blood of some of these animals was also collected and analyzed as previously described [17].

### Sample processing

200 μL of semen, or 200 μL of 10% w/v testes stroma or epididymis homogenate (prepared in PBS plus a cocktail of proteinase inhibitors (Roche)) were centrifuged at 100,000 x g for 1 hour and 4°C in the presence of 10% w/v sarkosyl. Supernatants were carefully discarded and undisrupted pellets centrifuged in PBS in the same conditions explained above for 30 minutes. Resulting pellets were resuspended in PMCA substrate and submitted to PMCA as explained below. Additional tissue extracts from CWD-negative animals or PBS (190 μL) were spiked with a serially diluted brain extract from an experimentally infected and symptomatic CWD-WTD (10 μL) and also submitted to PMCA.

### CWD prion detection by PMCA

PMCA substrate was prepared by homogenizing perfused brains of homozygous Tg1536 mice [18], as previously described [17]. Conversion buffer composition included 150 mM NaCl, 1% v/v Triton X-100, 6 mM EDTA and 0.025% v/v digitonin in PBS. PMCA substrates were prepared at 10% w/v and debris were removed by low speed centrifugation (805 x g, 45 seconds). Supernatants were mixed, aliquoted and stored at -80°C until use. Sample/PMCA-substrate mixtures were placed in a programmable sonicator (QSonica) and subjected to incubation/sonication cycles (29 minutes and 40 seconds incubation, 20 seconds sonication). Three or four PMCA rounds were performed for each sample as shown in Figures. First PMCA round consisted on 144 cycles (72 hours) and second, third and fourth rounds comprised 96 cycles (48 hours). Each specimen was tested in triplicate by two different investigators (C.K. and R. G-G). Samples were considered positive if at least two of the replicates showed positive signal. PrP^Sc^ signals generated by PMCA were assessed by western blot, as described [17,19], using PRC1 as primary antibody. Each set of PMCA data had at least five unseeded reactions, which acted as negative controls, and serially diluted CWD prions (10^−7^–10^−10^ brain dilutions) of known PMCA efficiency that acted as positive controls.

## Results

Samples analyzed in this study were obtained *post mortem* from 21 depopulated captive WTD bucks from farms across the United States. CWD-positive animals used in this study (n = 9) were pre-clinical but possessed immunohistochemistry (IHC) PrP^Sc^ staining in the obex and/or the medial retropharyngeal lymph nodes (MRPLN). Animals were classified as early or late in the disease course based on whether PrP^Sc^ IHC staining was present exclusively in the MRPLN (early) or in both the MRPLN and obex (late) (as previously described [17]). Samples from twelve CWD bucks negative for PrP^Sc^ by IHC were used as controls. The age range of the study animals was between one and seven years of age, and animals possessed different polymorphisms at position 96 of the prion protein (S1 Table). Testes were dissected into the testes stroma and epididymides, and were treated as individual specimens. PMCA manipulators were blinded to the CWD status of the samples until the experiments were completed.

**Fig 1:**
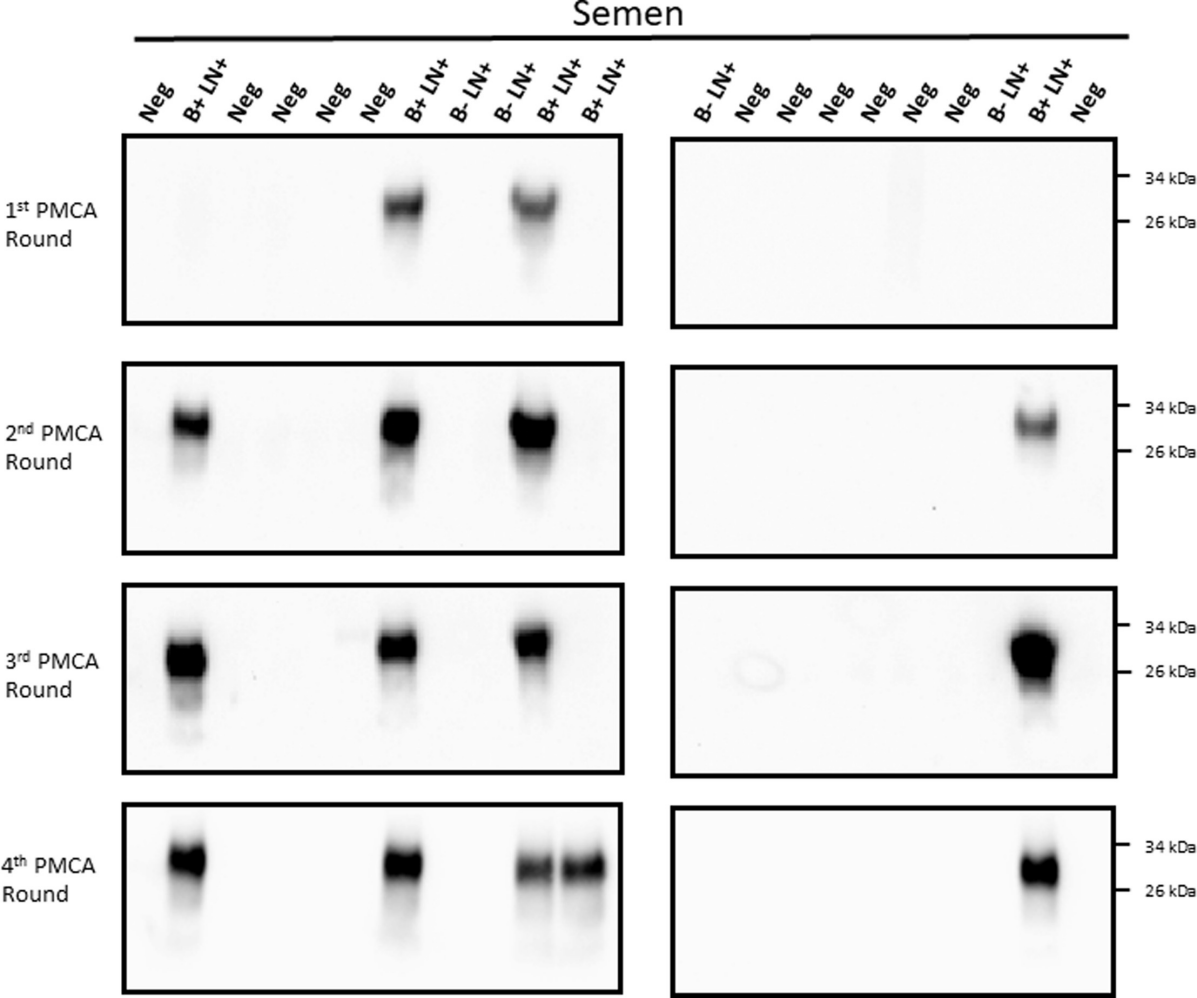
CWD prion detection in semen samples from white-tailed deer. Twenty-one semen samples from farmed white tailed-deer were tested for their presence of CWD prions by PMCA. Four serial PMCA rounds were performed to increase sensitivity. B+ LN+: had PrP^Sc^ deposition in the brain stem and medial retropharyngeal lymph nodes (MRPLN) by immunohistochemistry (late pre-symptomatic); B- LN+: had PrP^Sc^ deposition only in the MRPLN (early pre-symptomatic); Neg: samples were non-detect for CWD in both the brain stem and the MRPLN. Numbers at the right represent molecular weight markers.

**Fig 2:**
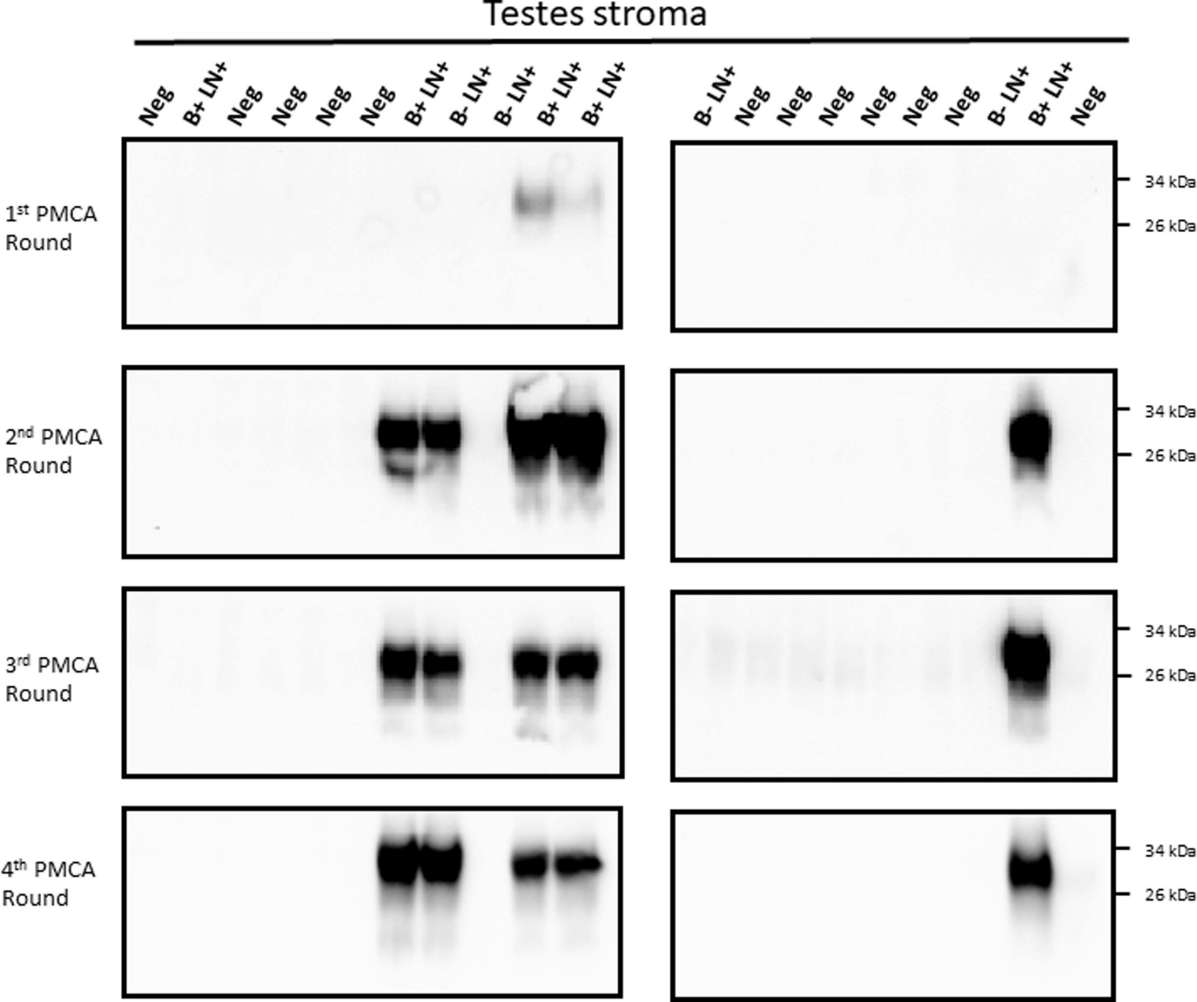
Evaluation of CWD prions presence in testes stroma of white-tailed deer bucks. Testes from twenty-one white tailed-deer (same animals depicted in Fig 1) were tested for the presence of CWD prions by PMCA. Similar methods as described in Fig 1 were used in this case. B+ LN+: had PrP^Sc^ deposition in the brain stem and medial retropharyngeal lymph nodes (MRPLN) by immunohistochemistry (late pre-symptomatic); B- LN+: had PrP^Sc^ deposition only in the MRPLN (early pre-symptomatic); Neg: samples were non-detect for CWD in both the brain stem and the MRPLN. Numbers at the right represent molecular weight markers.

**Fig 3:**
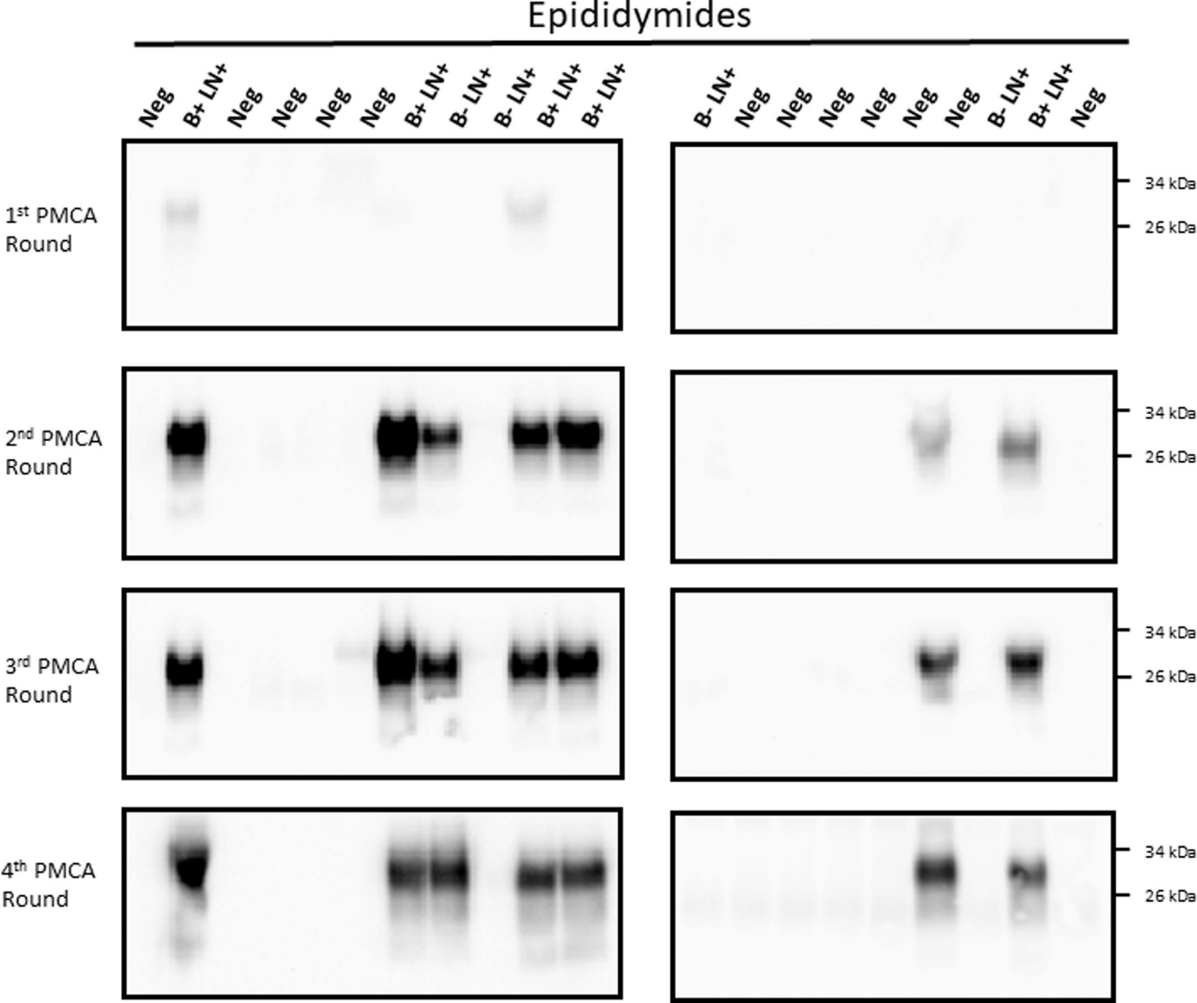
Identification of CWD prions in epididymides of white-tailed deer. Epididymides from white tailed-deer were analyzed for the presence of CWD prions by PMCA. Samples and methods utilized for detection in this case were the same as listed for Figs 1 and 2. B+LN+: had PrP^Sc^ deposition in the brain stem and medial retropharyngeal lymph nodes (MRPLN) by immunohistochemistry (late pre-symptomatic); B- LN+: had PrP^Sc^ deposition only in the MRPLN (early pre-symptomatic); Neg: samples were non-detect for CWD in both the brain stem and the MRPLN. Numbers at the right represent molecular weight markers.

For analyses, 200 μL of each specimen were concentrated before submitting to the PMCA procedure in order to increase detection odds. In the same line, four serial PMCA rounds were performed for each sample. The PMCA assay of semen samples detected PrP^Sc^ in 5/9 samples (55.5%, Fig 1). A similar detection efficiency was obtained for testes stroma (Fig 2), while epididymis detection increased to 66.6% (6/9 samples, Fig 3). All IHC negative animal samples were also negative by PMCA in the semen and testes. One of the 12 epididymides samples obtained from IHC-negative deer repeatedly came up as positive by PMCA (Fig 3). Contamination at the PMCA sample preparation level was unlikely as repeated sample preparation using prion-free materials always derived in the same result. Overall, the combined sensitivity of detection for all samples tested reached 59.3% and a specificity of 97.2% (Table 1).

**Table 1:** Overall CWD prion detection reproductive tissues and semen from white-tailed deer bucks.

Biological Sample	Sensitivity	Specificity
Semen	55.5% (5/9)	(0/12) 100%
Testes	55.5% (5/9)	100% (0/12)
Epididymides	66.6% (6/9)	91.6% (1/12)
Overall	59.3%	97.2%

Samples were declared as positive in PMCA if at least two of the triplicates gave a protease-resistant PrP^Sc^ signal in any of the PMCA rounds analyzed.

We further analyzed the distribution of CWD-positive samples using different parameters, including PrP polymorphisms, disease stage and age. S1 Table display specific data for the animals used in this study. Our results showed 100% detection (5/5) for semen and epididymis in animals at late pre-clinical stages (Table 2). Similar results were obtained for testes stroma where 80% detection was achieved on this group of animals. In contrast, detection was poor for animals in the early pre-clinical stage, with no detection in semen, and 25% in testes and epididymides.

**Table 2:** Detection of CWD prions male reproductive tissues and semen from white-tailed deer at different incubation periods.

Biological Sample	Late pre-symptomatic	Early pre-symptomatic
Semen	100% (5/5)	0% (0/4)
Testes	80% (4/5)	25% (1/4)
Epididymides	100% (5/5)	25% (1/4)
Overall	93.3% (14/15)	16.6% (2/12)

Samples were declared as positive in PMCA if at least two of the triplicates gave a protease-resistant PrP^Sc^ signal in any of the PMCA rounds analyzed.

We previously communicated that blood detection by PMCA reached sensitivity levels of ~96% at late pre-symptomatic stages [17]. Considering the high levels of detection we obtained in semen at late stages of the CWD incubation period, we tested both blood and semen samples from six CWD infected bucks. Four of the six animals tested positive for CWD in both the semen and the blood, while the remaining two were negative in both (Figs 1 and S1). Blood and semen from a CWD negative animal were used to confirm specificity. These results suggest that PrP^Sc^ distribution in semen and blood of CWD infected bucks may be similar at late incubation periods.

**Fig 4:**
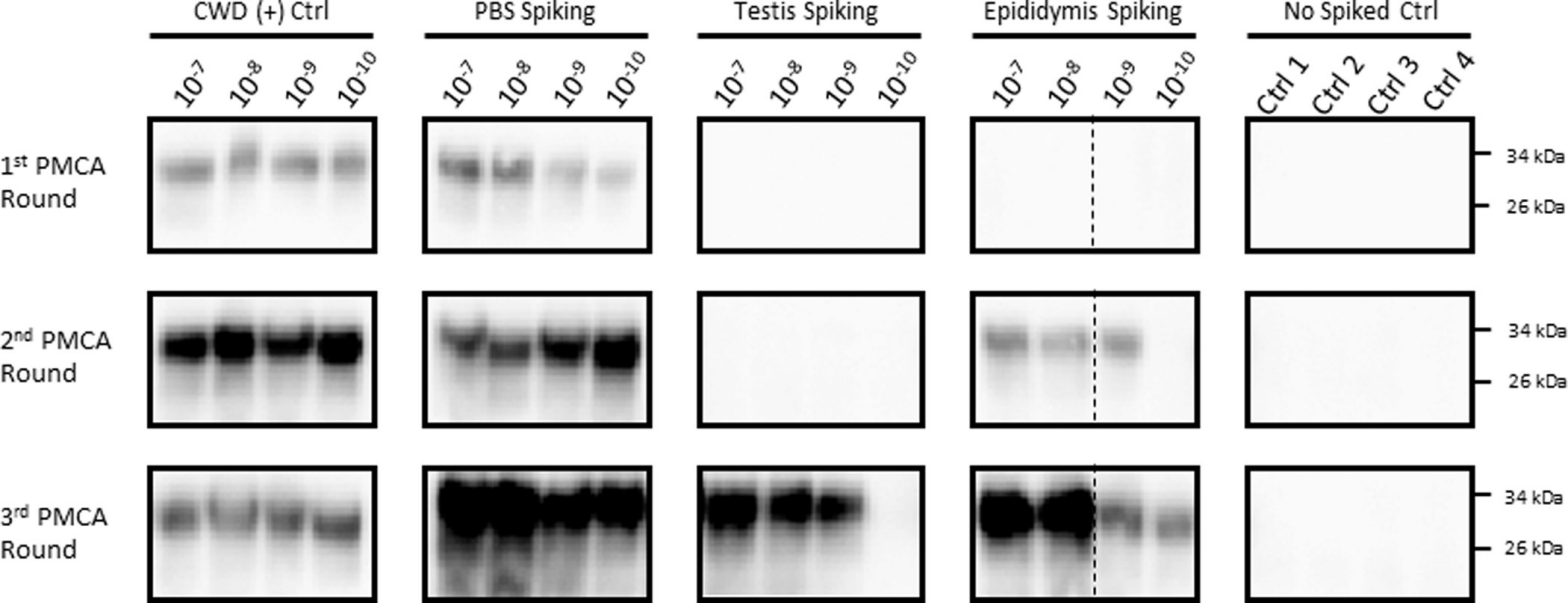
Modulation of PMCA efficiency by testes and epididymides homogenates. Known amounts of brain-derived CWD prions (10^−7^–10^−10^ brain dilutions) were spiked in testis and epididymis homogenates from a CWD-negative white tailed-deer. Samples were processed in the same way as samples depicted in previous figures and tested in three PMCA rounds. Positive controls included samples spiked in PBS, or directly spiked in PMCA substrate (CWD (+) Ctrl). Negative controls comprised unseeded reactions (Ctrl1-4). Numbers at the right represent molecular weight markers. Dotted lines represent splicing of different membranes.

The results presented above clearly show that sexual tissues and fluids from WTD bucks contain detectable levels of CWD prions at pre-symptomatic stages of the disease. However, it is still unclear whether these samples contain enough quantities of PrP^Sc^ to sustain disease transmission. Careful analysis of the PMCA data suggests similar efficiencies of prion detection for individual samples, as most of them provided PrP^Sc^ signals at the second PMCA round (Figs 1–3). However, our extensive experience with PMCA has taught us that PMCA performance can be affected when using samples other than brain extract and purified proteins [17,19–25]. Importantly, this could result in underestimation of PrP^Sc^ quantities. To test the performance/efficiency of PMCA in the reproductive tissues of male deer, we spiked testis and epididymis homogenates from a CWD negative deer with different dilutions of brain-derived CWD prions (Fig 4). Results show that spiking with CWD prions in PBS and further processing of the sample (as explained in “Materials and methods”) did not affect PMCA efficiency when compared to spiking directly with infected brain extracts (Fig 4, left panels). In both cases, maximum amplification reached 1 x 10^−10^ CWD infected brain dilution in the first PMCA round. As expected, testis stroma and epididymis affected PMCA performance. Testis homogenate displayed negative prion detection in the first two PMCA rounds, rendering positive PrP^Sc^ signals only at the third round, albeit with a lower maximum efficiency when compared to the positive control. Epididymis homogenate also showed inhibition for *in vitro* prion replication, although in this case, the effect was considerably lower when compared to testes. Negative results in unseeded PMCA reactions confirmed the specificity of the assay. Unfortunately, similar experiments using semen were not possible due to insufficient sample volume.

Polymorphic variation in WTD has been described as an important factor mediating susceptibility for CWD [26–29], as well as PrP^Sc^ tissue distribution and shedding [11]. In the current experiment, samples from bucks carrying different PrP polymorphisms at position 96 were tested. Prion detection in different samples did not appear affected by PrP polymorphic variation (Table 3). We also assessed whether CWD detection by PMCA was related with age. Our results showed no correlation between these two parameters, suggesting that animals used in this study were exposed to CWD prions at different stages of their lives. Unfortunately, sample size was too small to provide convincing evidence on whether PrP polymorphic variation or age played a role in the tropism of prions towards reproductive tissues.

**Table 3:** CWD prion detection in male reproductive tissues and semen from white-tailed deer depending of prion protein polymorphic variation at position 96.

Biological Sample	96GG	96 GS	96 SS
Semen	3/5	1/2	1/2
Testes	2/5	1/2	2/2
Epididymides	3/5	1/2	2/2
Overall	53.5% (8/15)	50% (3/6)	83.3% (5/6)

Samples were declared as positive in PMCA if at least two of the triplicates gave a protease-resistant PrP^Sc^ signal in any of the PMCA rounds analyzed.

## Discussion

Our results show, for the first time, the presence of CWD prions in semen and reproductive tissues of CWD-infected WTD bucks. Our results are relevant considering that samples were obtained from naturally infected animals, in which prion incubation periods and routes of exposure are unknown. These results shed light on PrP^sc^ trafficking within the body and indicate that live animal studies are warranted to understand the potential role of semen in CWD transmission. From the detection perspective, our results show a similar degree of efficiency when compared to blood detection using similar methods. Overall detection in CWD positive samples was 59.3%. This ratio increased to 93.3% when only samples from the late CWD pre-clinical stage were considered. Prion detection in testes stroma and epididymides behaved similarly as to semen, suggesting that prion tropism to male sexual related tissues and fluids occurs only at late stages. It is important to note that sample size for this study was considerably lower compared to the previous study evaluating blood samples [17].

Considering our previous data on CWD prion detection in blood [17], it is valid to question whether our positive PMCA results in male reproductive tissues and semen are due to this fluid. It is important to mention that the semen samples used in this study did not have noticeable blood contamination by visual examination. With regard to testes and epididymis, the presence of blood is undeniable. Nevertheless, and as described in Fig 4, components present in these samples strongly inhibit PMCA performance and negate the possible effect of blood in our results.

Some unexpected results were observed in this study (S1 Table). One of them involves one animal at late CWD pre-symptomatic stage that was PMCA positive in semen and epididymis, but negative in testes. Another involves an early pre-symptomatic animal that was CWD positive in testis and epididymis, but negative in semen. This could be explained by the low presence of PrP^Sc^ in these samples and the inhibition that testes and epididymis homogenates exerted in PMCA (Fig 4). These two factors could play an important role in our PMCA readouts. In that sense, future analyses of additional and higher number of samples, and tested in multiple replicates, could tell us whether these off-trend cases are caused by technical issues related to the PMCA technology. Another explanation for this behavior could be found in the strain variation displayed by CWD prions [27,29–31]. Prion strains are known to exert different pathological and clinical features in the host, including different tissue tropism [32]. Considering that our samples were obtained from natural CWD cases, it could be possible that animals were infected with prion isolates targeting the reproductive system of bucks in different patterns. This idea acquire additional support in the fact that animals included in this study displayed different polymorphic versions of the prion protein (an additional source of prion strain variation[33]). Another unexpected result came from one animal in the CWD-free group that resulted in positive PrP^Sc^ detection in epididymis. The simplest explanation for this result is cross-contamination. Although samples were carefully collected to avoid contamination, this is always a possibility when working with field collected specimens. Another possibility is that our PMCA format detected prions in these samples earlier than IHC in lymph nodes. The contribution of all the previously mentioned variables in sexual tissue–PrP^Sc^ detection should be pursued in carefully designed future studies.

In terms of disease transmission, the presence of prions in semen begs the question on whether sexual contact is plausible route of CWD transmission. A previous report showed that semen collected from rams at pre-clinical and clinical stages of prion disease did not infect scrapie-susceptible mice [34]. Our previous results in Syrian hamsters showed that sexual exposure of naïve females to 263K infected males was ineffective in transmitting disease [35]. Maternal transmission has also be presented as a viable mode of CWD transmission to offspring. Evidence derived from scrapie-infected sheep and experimentally infected muntjac deer provides direct evidence that offspring from infected dams and ewes are at higher risk of developing prion disease [16,36]. Considering the results presented in this article, the risk of CWD transmission via semen cannot be dismiss without further inquiry. It is important to note that some semen samples tested in the current report showed PrP^Sc^ presence after only one PMCA round, suggesting that PrP^Sc^ content in semen of some animals may be relatively high. This is particularly relevant considering that tissues from male sexual organs inhibited PMCA performance. It remains unclear if vaginal exposure to CWD prions in semen is an effective route of transmission.

In summary, our results confirm the presence of CWD prions in semen and male sexual tissues in CWD-infected WTD. Future experiments in actual deer will determine whether CWD can be transmitted by breeding practices including sexual contacts or artificial inseminations. Infectivity studies in transgenic mice underway in our laboratory will determine the infectivity titers of some of the samples described in this study.

## Supporting information

S1 Fig CWD prion detection in blood of white-tailed deer. Seven whole blood samples from white tailed-deer were tested for their presence of CWD prions by PMCA. **A)** Results depicting PrP^Sc^ presence at the fourth PMCA round. B+ LN+: sample positive for PrP^Sc^ deposition at brain stem and lymph nodes by immunohistochemistry (late pre-symptomatic); B- LN+: sample positive for PrP^Sc^ deposition at lymph nodes only (early pre-symptomatic); Neg: CWD-negative samples. Numbers at the right represent molecular weight markers. Dotted lines represent splicing of different membranes. **B)** Summary table comparing CWD prion detection in semen and blood samples.
(TIF)

S2 Fig Uncropped pictures used in western blots (Figs 1–4). The series of pictures presented here has the purpose to show raw data from the western blots used in this article.
(PDF)

S3 Fig Uncropped pictures used in western blots (S1 Fig). The series of pictures presented here has the purpose to show raw data from the western blots used in this article.
(PDF)

S1 Table Individual data of white tail deer bucks used in this study. (PPTX)
