# Supplementary figures and images for: In Vitro detection of Chronic Wasting Disease (CWD) prions in semen and reproductive tissues of white tailed deer bucks (Odocoileus virginianus)
Source: PLoS One. 2019 Dec 30;14(12):e0226560. doi: 10.1371/journal.pone.0226560 (PMC6936793; doi:10.1371/journal.pone.0226560)

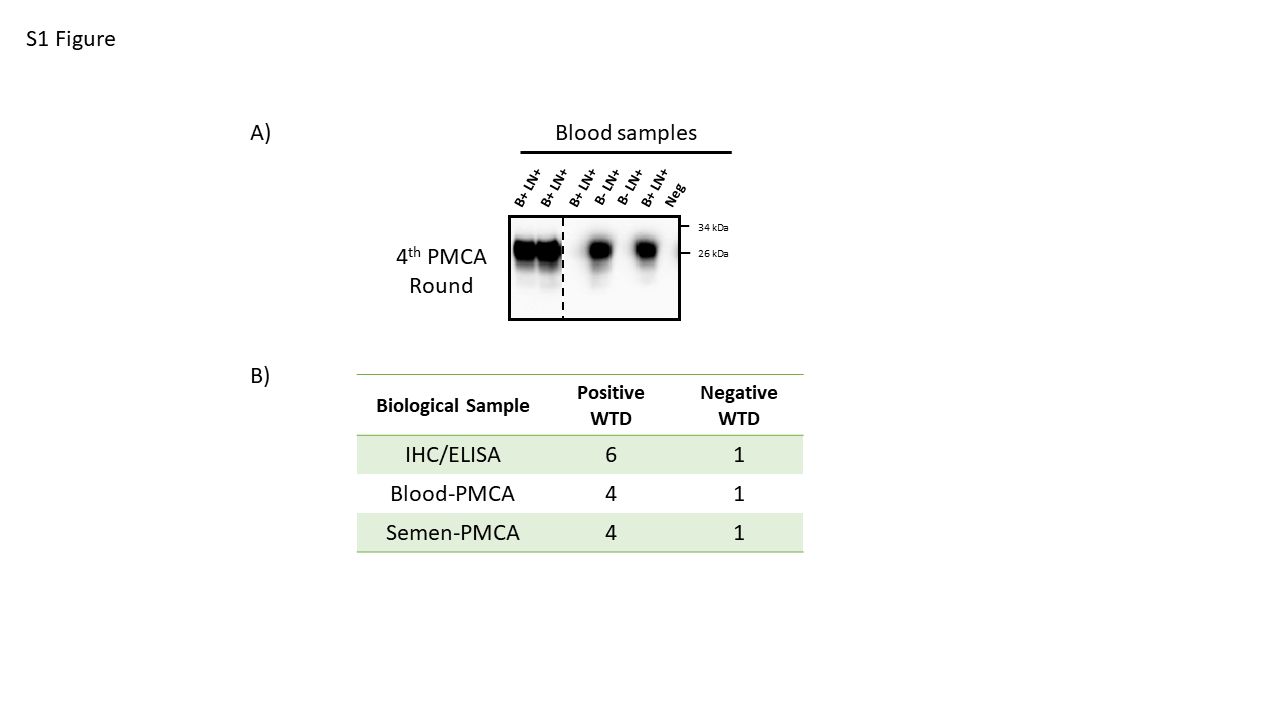

Supplement: S1 Fig — Seven whole blood samples from white tailed-deer were tested for their presence of CWD prions by PMCA. A) Results depicting PrPSc presence at the fourth PMCA round. B+ LN+: sample positive for PrPSc deposition at brain stem and lymph nodes by immunohistochemistry (late pre-symptomatic); B- LN+: sample positive for PrPSc deposition at lymph nodes only (early pre-symptomatic); Neg: CWD-negative samples. Numbers at the right represent molecular weight markers. Dotted lines represent splicing of different membranes. B) Summary table comparing CWD prion detection in semen and blood samples. (TIF) [file pone.0226560.s001.tif]

S1 Figure

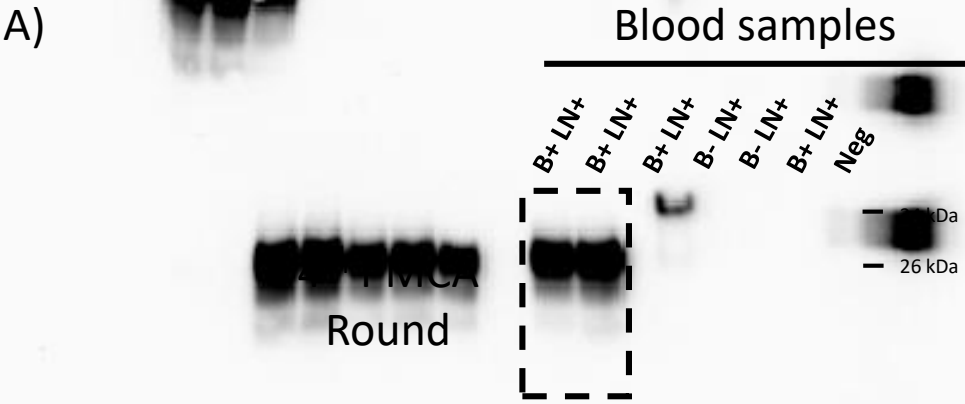

# S1 Figure

A)

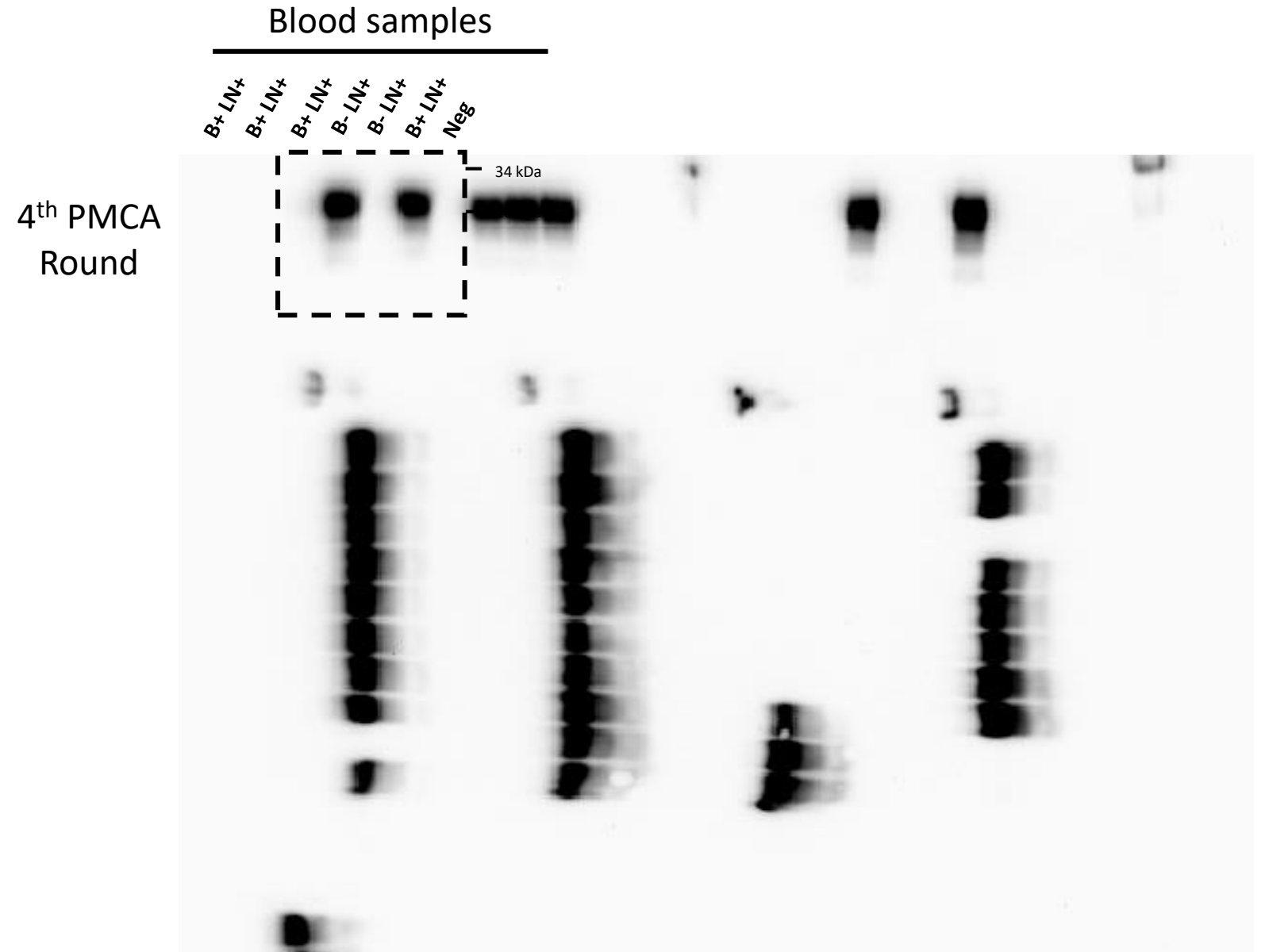

Supplement: S3 Fig — The series of pictures presented here has the purpose to show raw data from the western blots used in this article. (PDF) [file pone.0226560.s003.pdf]
