# Supplementary material for: In Vitro detection of Chronic Wasting Disease (CWD) prions in semen and reproductive tissues of white tailed deer bucks (Odocoileus virginianus)
Source: PLoS One. 2019 Dec 30;14(12):e0226560. doi: 10.1371/journal.pone.0226560 (PMC6936793; doi:10.1371/journal.pone.0226560)
Supplement: S2 Fig — The series of pictures presented here has the purpose to show raw data from the western blots used in this article. (PDF) [file pone.0226560.s002.pdf]

Figure 1

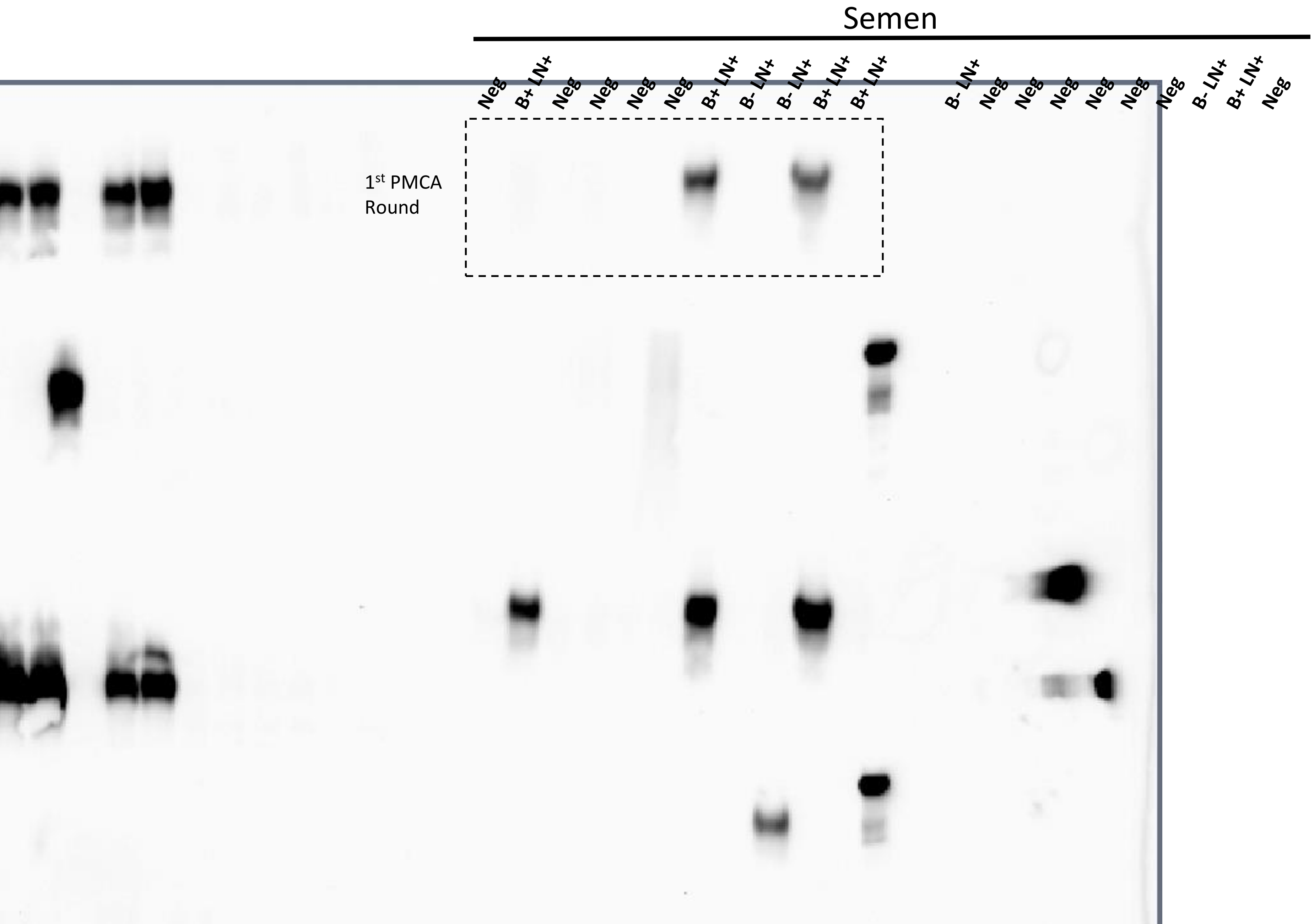
Semen
Neg
B+ LN+
Neg
Neg
Neg
Neg
B+ LN+
B- LN+
B- LN+
B+ LN+
B+ LN+
B- LN+
Neg
Neg
Neg
Neg
Neg
Neg
B- LN+
B+ LN+
Neg
1st PMCA Round

Figure 1

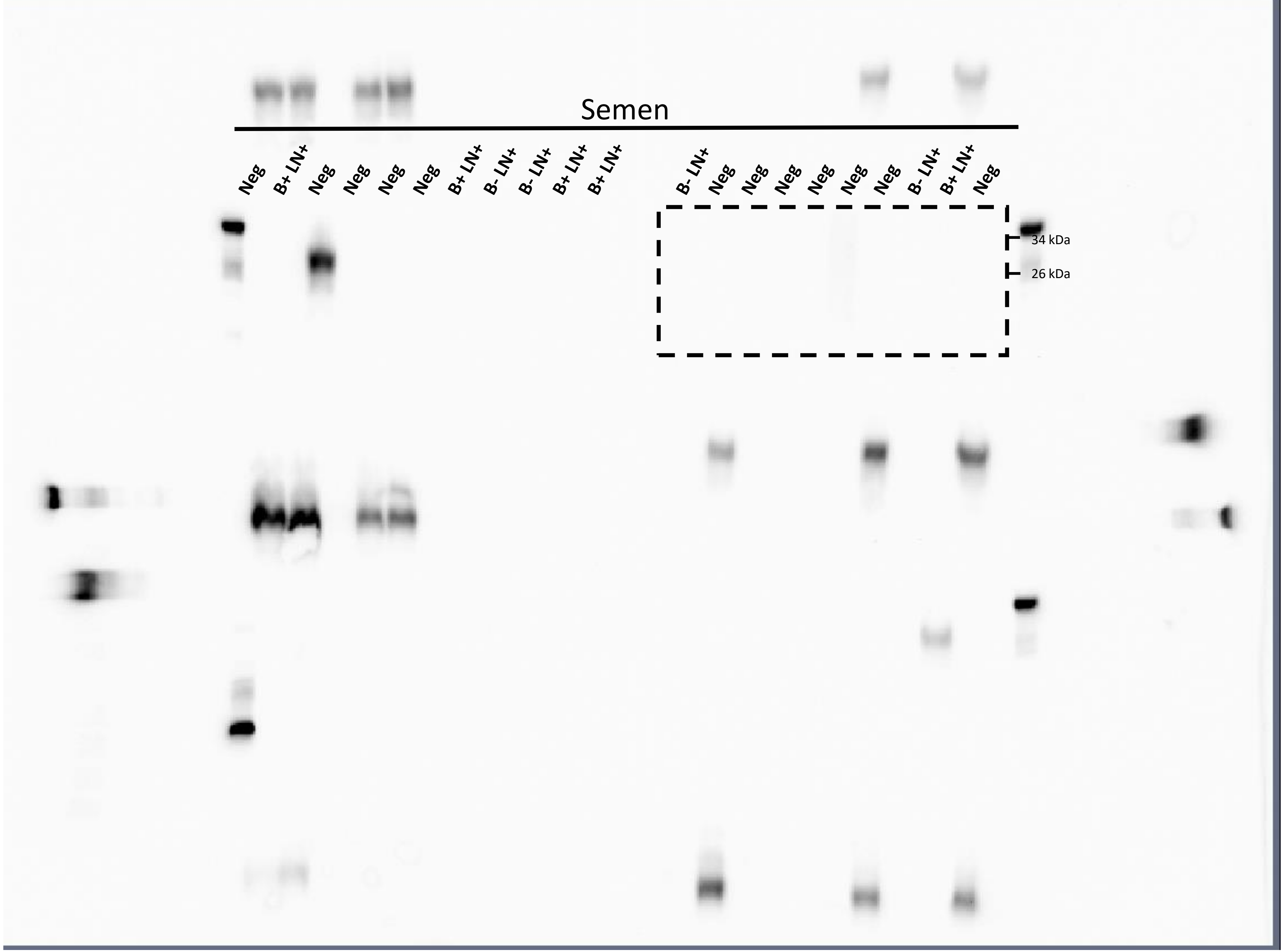
Semen
Neg
B+ LN+
Neg
Neg
Neg
Neg
B+ LN+
B- LN+
B- LN+
B+ LN+
B+ LN+
B- LN+
Neg
Neg
Neg
Neg
Neg
Neg
B- LN+
B+ LN+
Neg
34 kDa
26 kDa

Figure 1

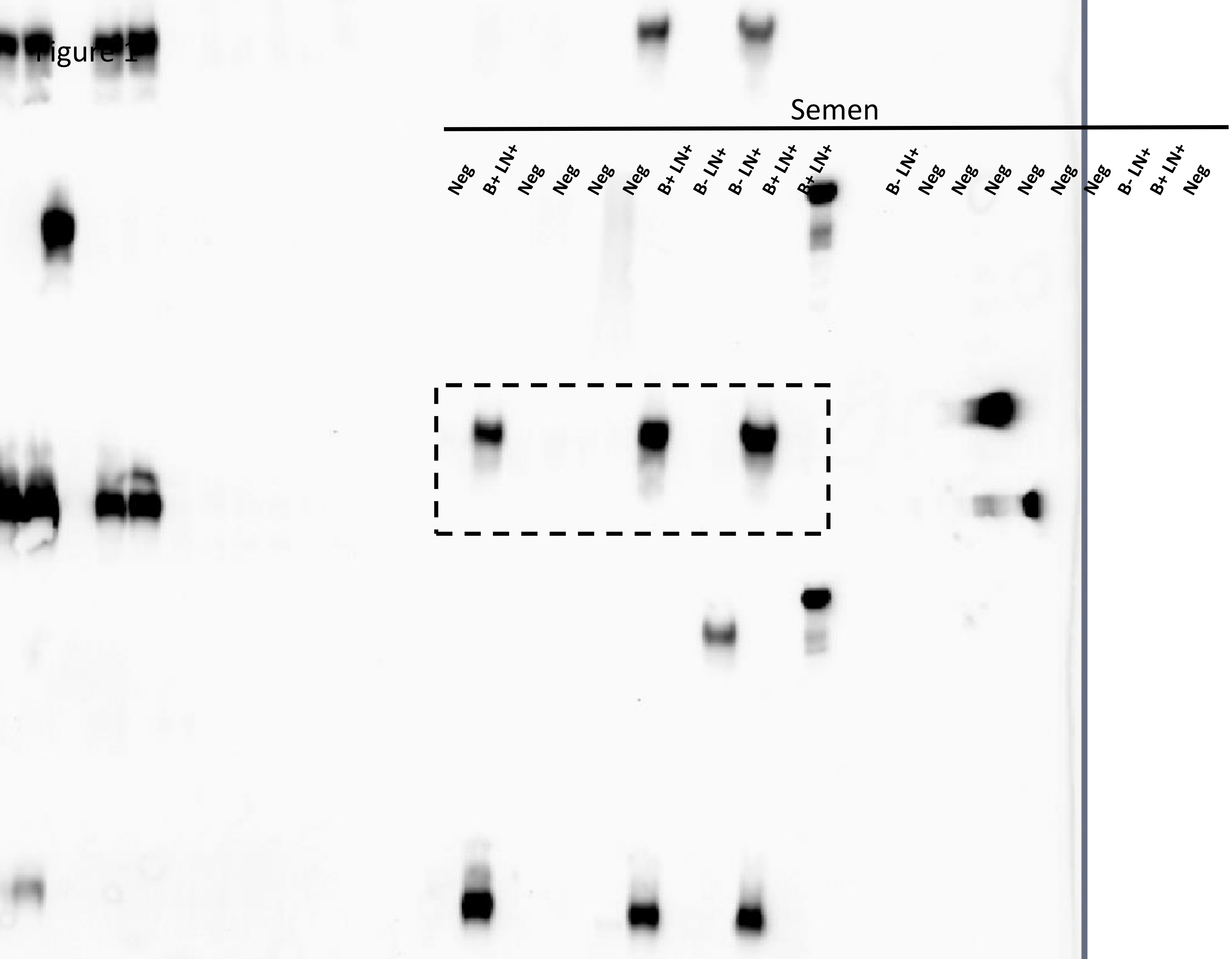
Semen
Neg
B+ LN+
Neg
Neg
Neg
Neg
B+ LN+
B- LN+
B- LN+
B+ LN+
B+ LN+
B- LN+
Neg
Neg
Neg
Neg
Neg
Neg
B- LN+
B+ LN+
Neg

Figure 1

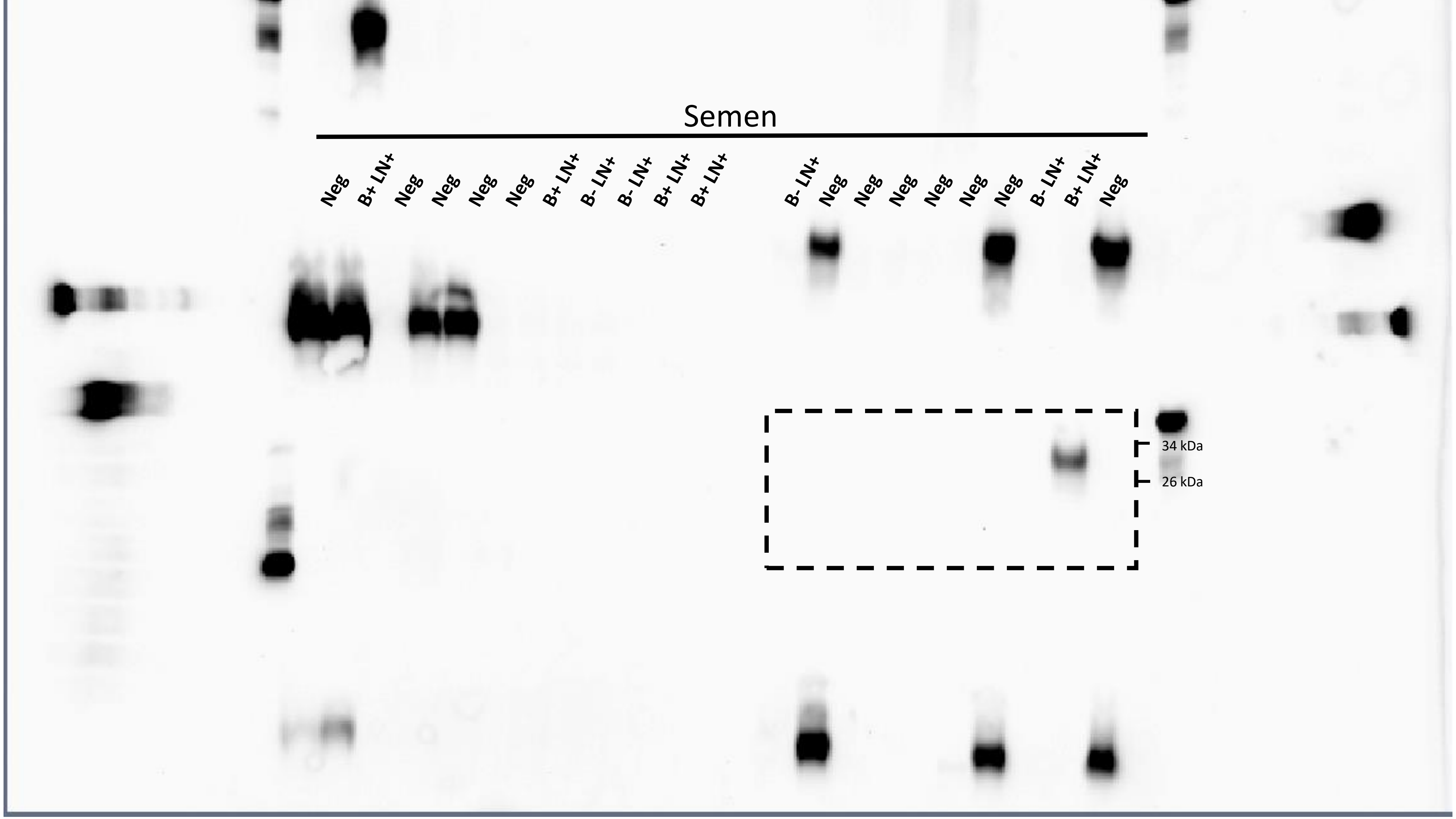
Semen
Neg
B+ LN+
Neg
Neg
Neg
Neg
B+ LN+
B- LN+
B- LN+
B+ LN+
B+ LN+
B- LN+
Neg
Neg
Neg
Neg
Neg
Neg
B- LN+
B+ LN+
Neg
34 kDa
26 kDa

Figure 1

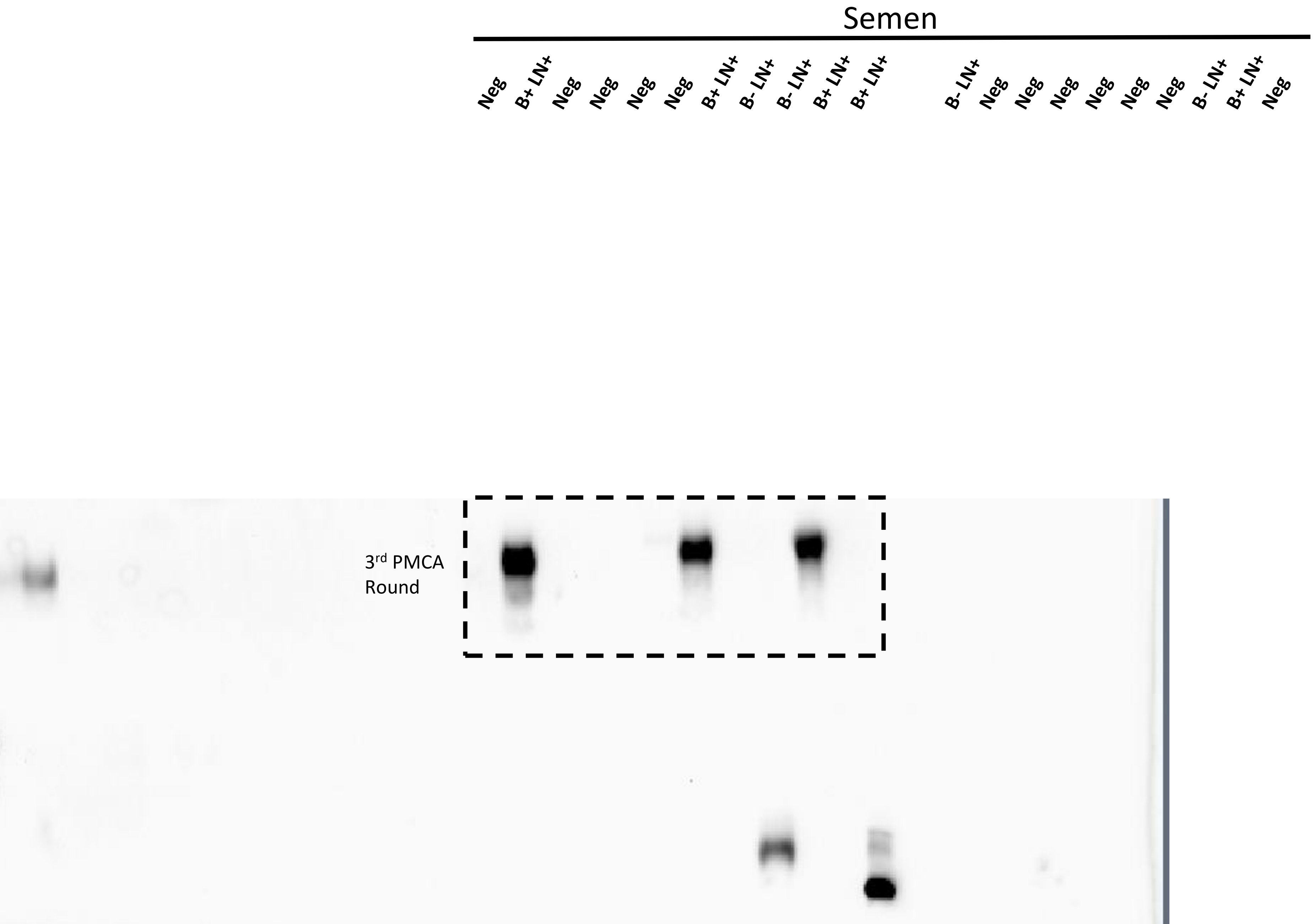
Semen
Neg
B+ LN+
Neg
Neg
Neg
Neg
B+ LN+
B- LN+
B- LN+
B+ LN+
B+ LN+
B- LN+
Neg
Neg
Neg
Neg
Neg
Neg
B- LN+
B+ LN+
Neg
3rd PMCA Round

Figure 1

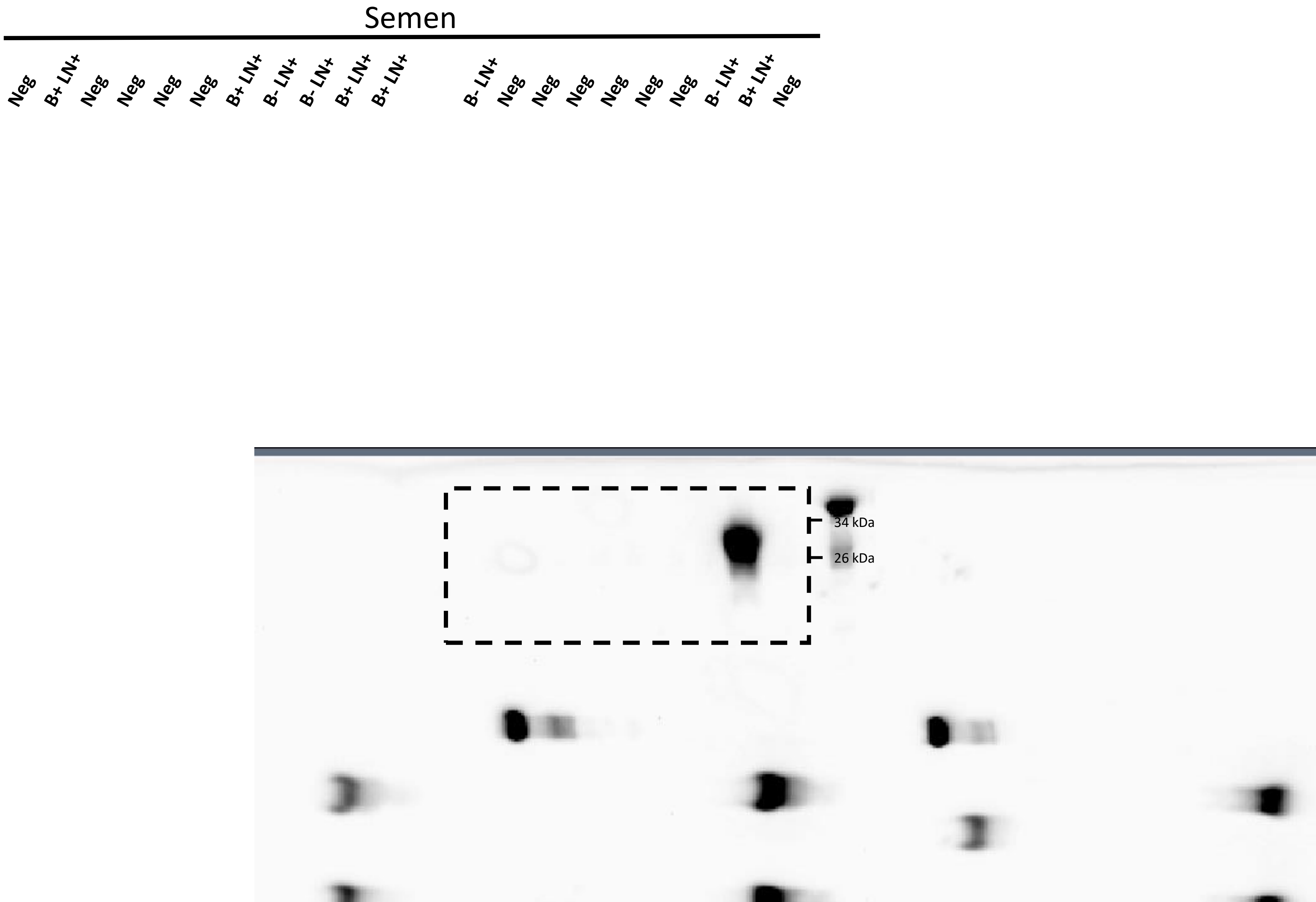
Semen
Neg
B+ LN+
Neg
Neg
Neg
Neg
B+ LN+
B- LN+
B- LN+
B+ LN+
B+ LN+
B- LN+
Neg
Neg
Neg
Neg
Neg
Neg
B- LN+
B+ LN+
Neg
34 kDa
26 kDa

Figure 1

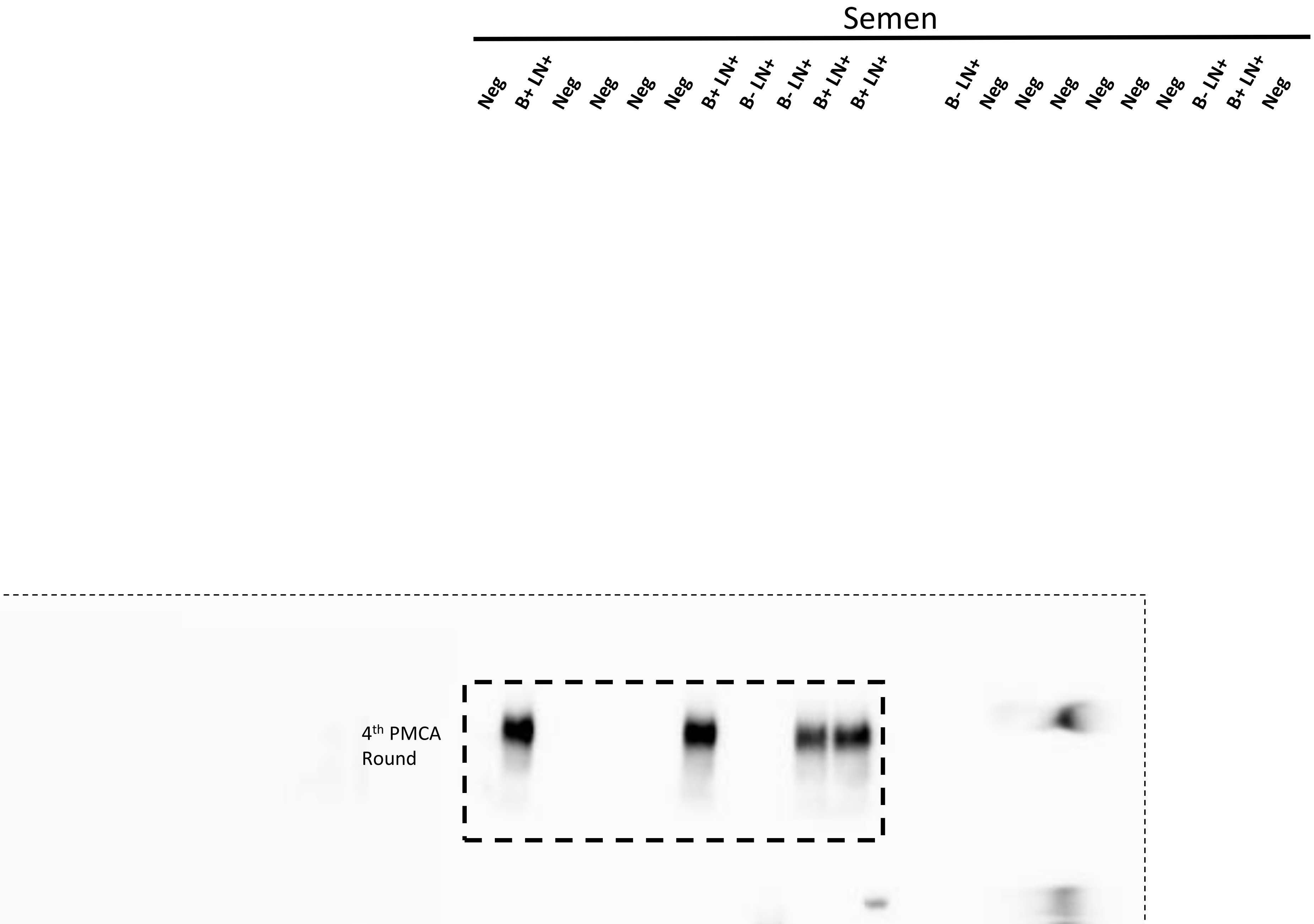
Semen
Neg
B+ LN+
Neg
Neg
Neg
Neg
B+ LN+
B- LN+
B- LN+
B+ LN+
B+ LN+
B- LN+
Neg
Neg
Neg
Neg
Neg
Neg
B- LN+
B+ LN+
Neg
4th PMCA Round

Figure 1

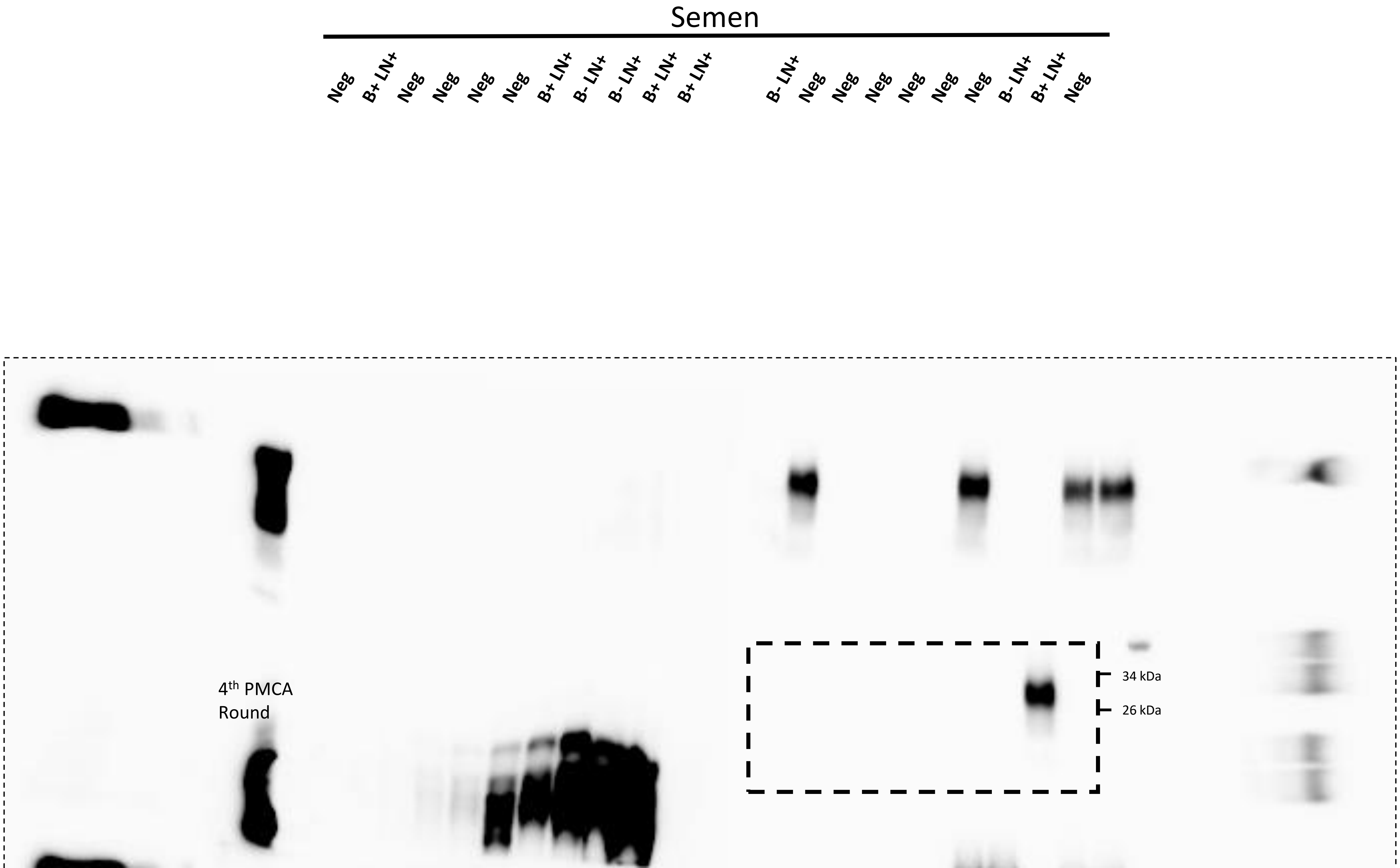
Semen
Neg
B+ LN+
Neg
Neg
Neg
Neg
B+ LN+
B- LN+
B- LN+
B+ LN+
B+ LN+
B- LN+
Neg
Neg
Neg
Neg
Neg
Neg
B- LN+
B+ LN+
Neg
4th PMCA Round
34 kDa
26 kDa

Figure 2

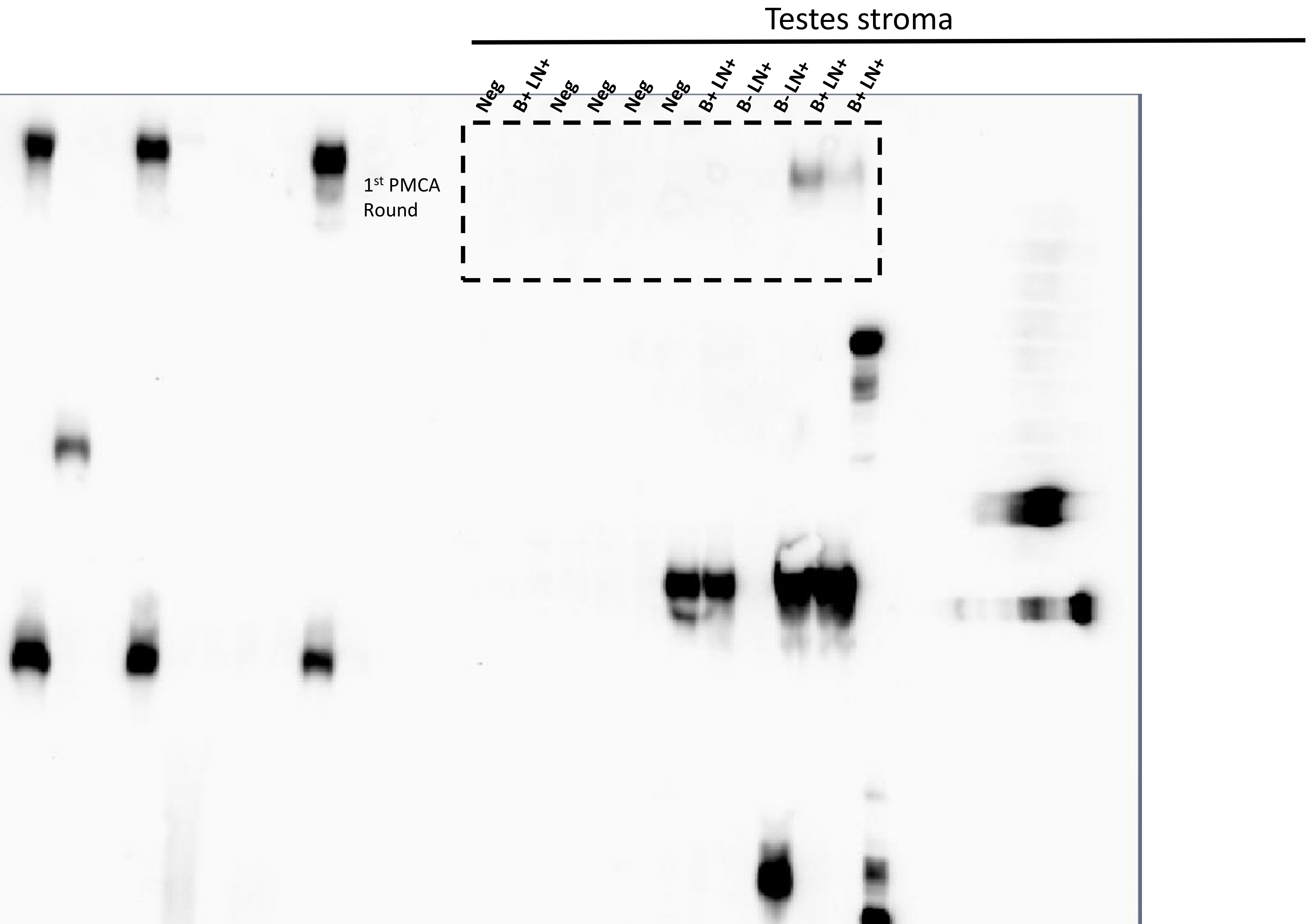

Testes stroma
Neg
B+ LN+
Neg
Neg
Neg
Neg
B+ LN+
B- LN+
B- LN+
B+ LN+
B+ LN+
1st PMCA Round

Figure 2

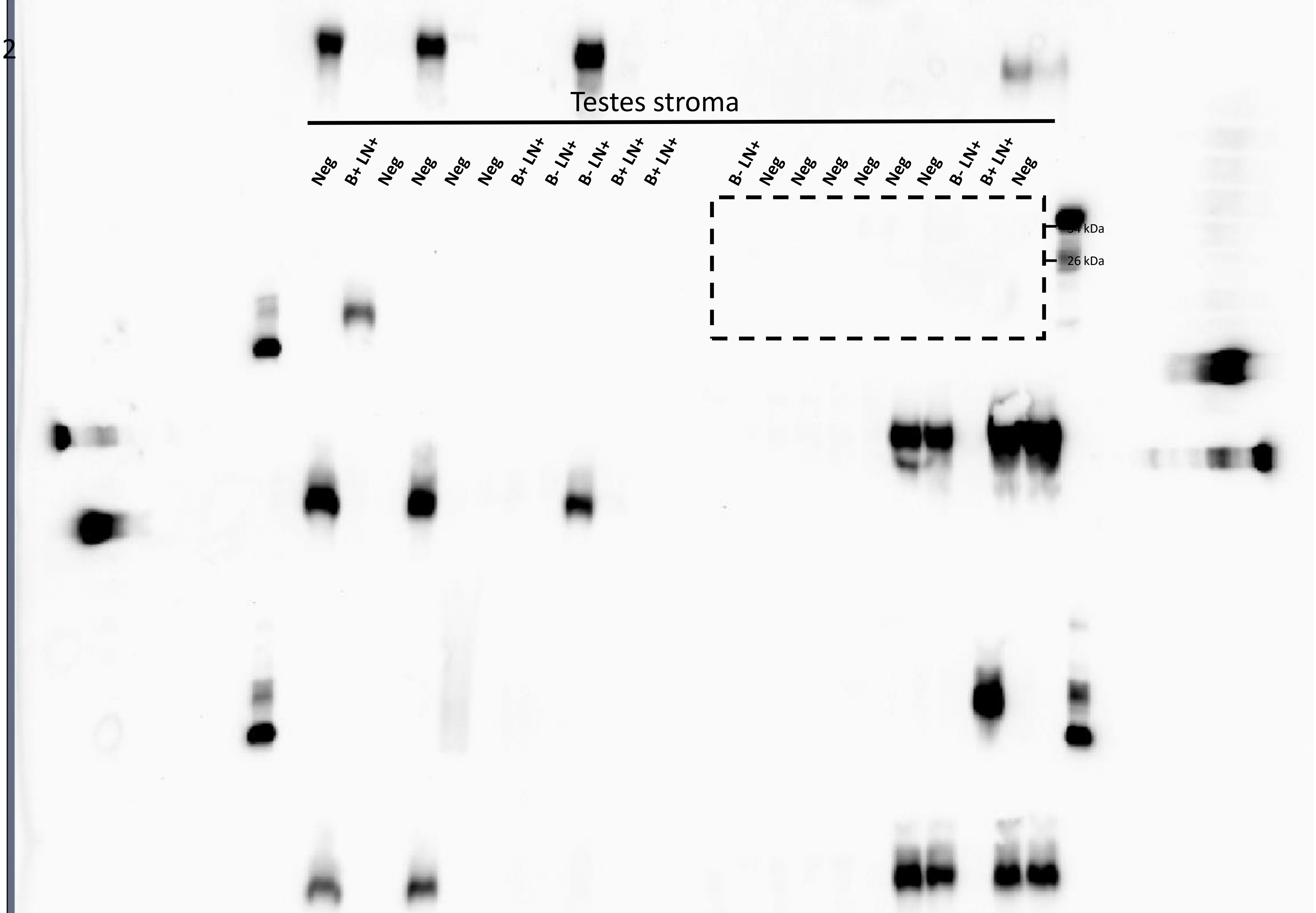
Testes stroma
Neg
B+ LN+
Neg
Neg
Neg
Neg
B+ LN+
B- LN+
B- LN+
B+ LN+
B+ LN+
B- LN+
Neg
Neg
Neg
Neg
Neg
Neg
B- LN+
B+ LN+
Neg
kDa
26 kDa

Figure 2

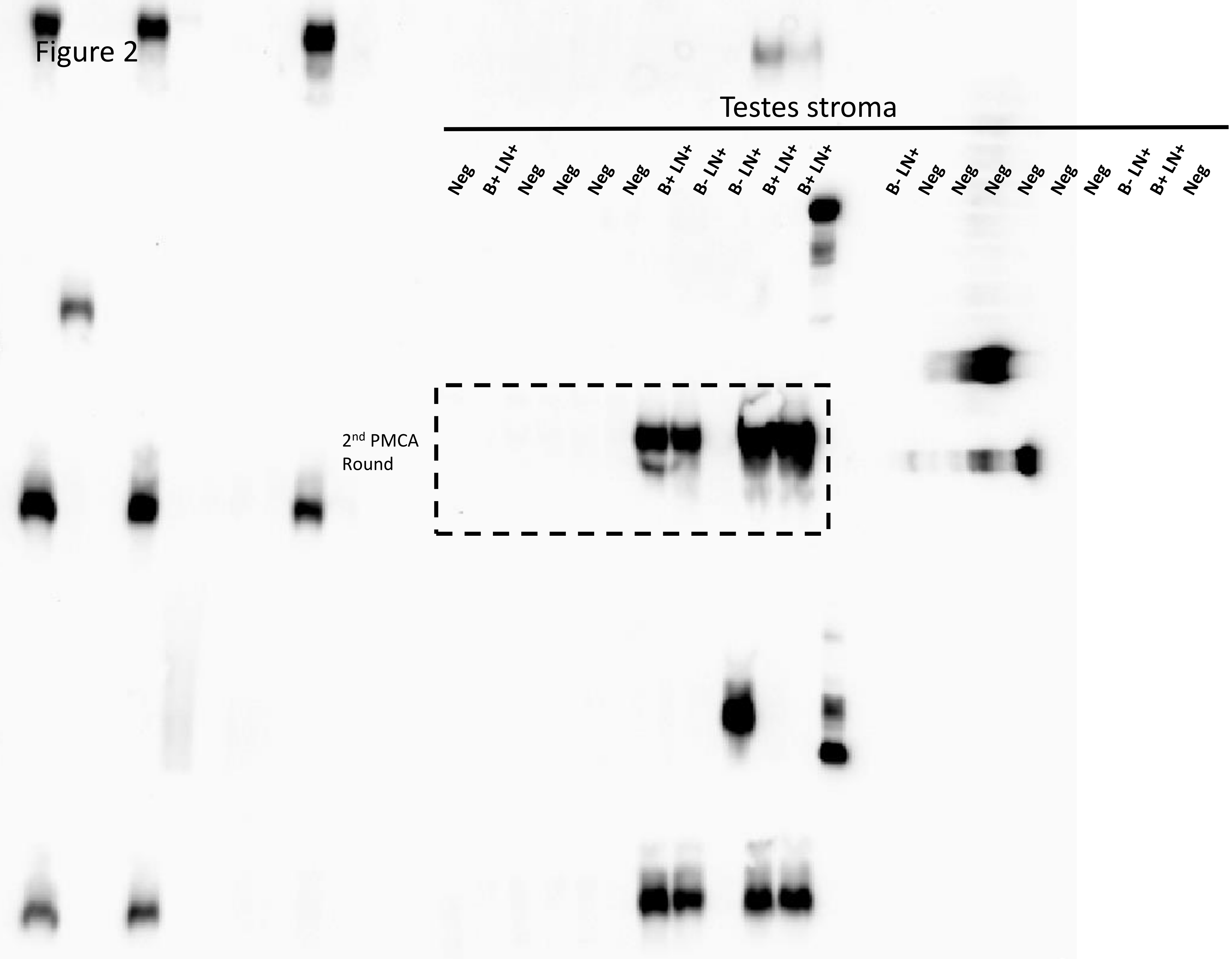
Testes stroma
Neg
B+ LN+
Neg
Neg
Neg
Neg
B+ LN+
B- LN+
B- LN+
B+ LN+
B+ LN+
B- LN+
Neg
Neg
Neg
Neg
Neg
Neg
B- LN+
B+ LN+
Neg
2nd PMCA Round

Figure 2

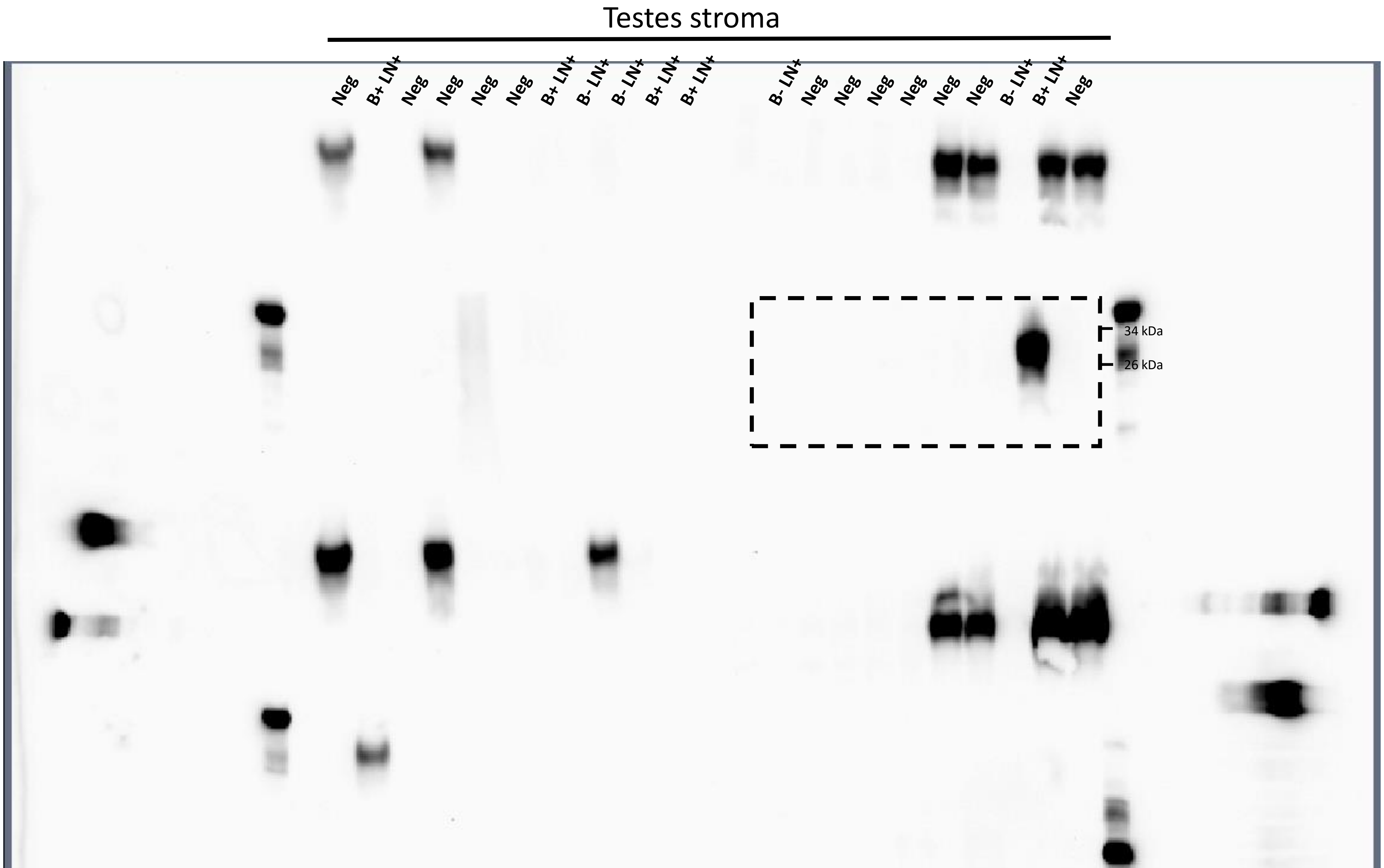
Testes stroma
Neg
B+ LN+
Neg
Neg
Neg
Neg
B+ LN+
B- LN+
B- LN+
B+ LN+
B+ LN+
B- LN+
Neg
Neg
Neg
Neg
Neg
Neg
B- LN+
B+ LN+
Neg
34 kDa
26 kDa

Figure 2

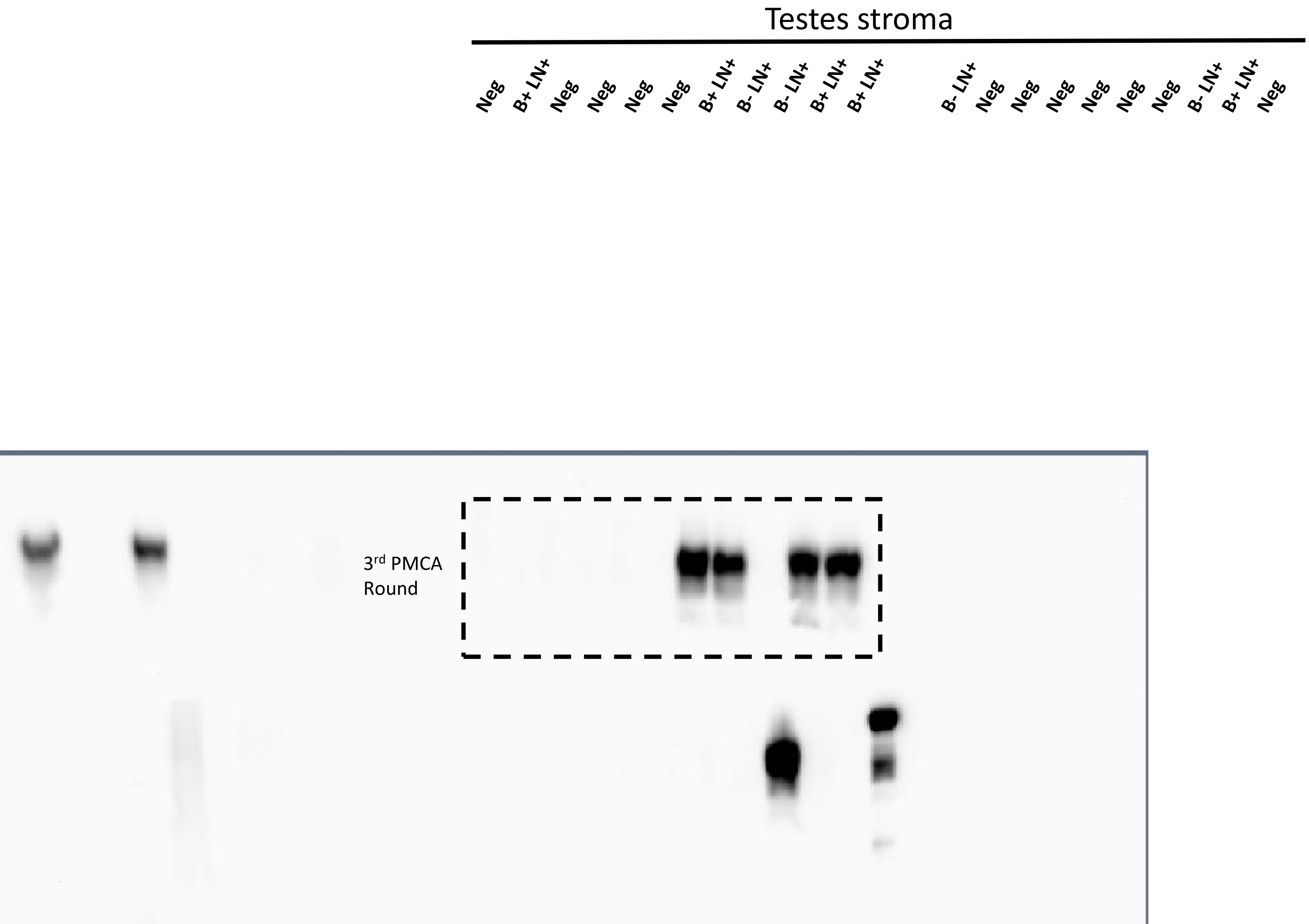
Testes stroma
Neg
B+ LN+
Neg
Neg
Neg
Neg
B+ LN+
B- LN+
B- LN+
B+ LN+
B+ LN+
B- LN+
Neg
Neg
Neg
Neg
Neg
Neg
B- LN+
B+ LN+
Neg
3rd PMCA Round

Figure 2

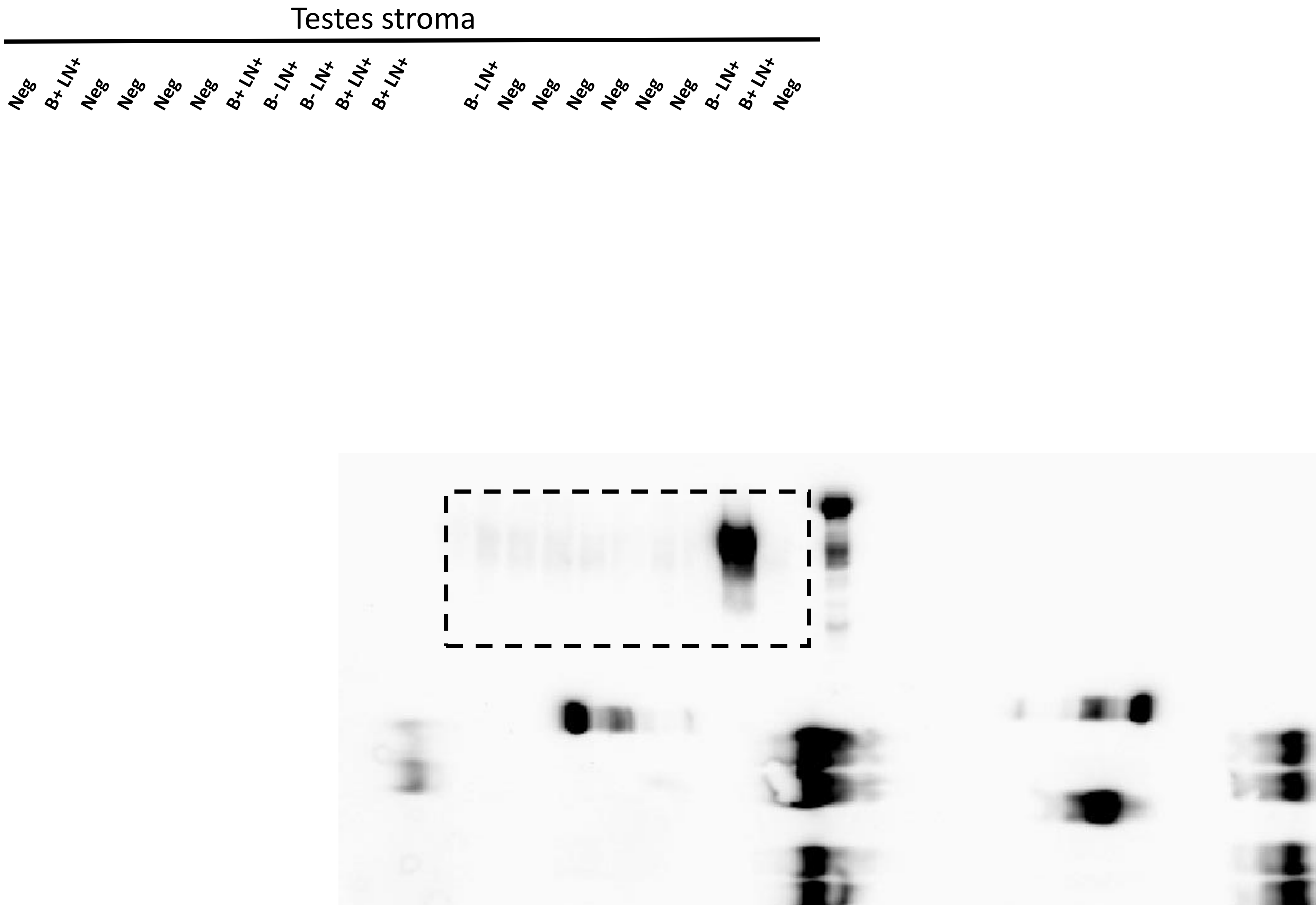

Testes stroma
Neg
B+ LN+
Neg
Neg
Neg
Neg
B+ LN+
B- LN+
B- LN+
B+ LN+
B+ LN+
B- LN+
Neg
Neg
Neg
Neg
Neg
Neg
B- LN+
B+ LN+
Neg

Figure 2

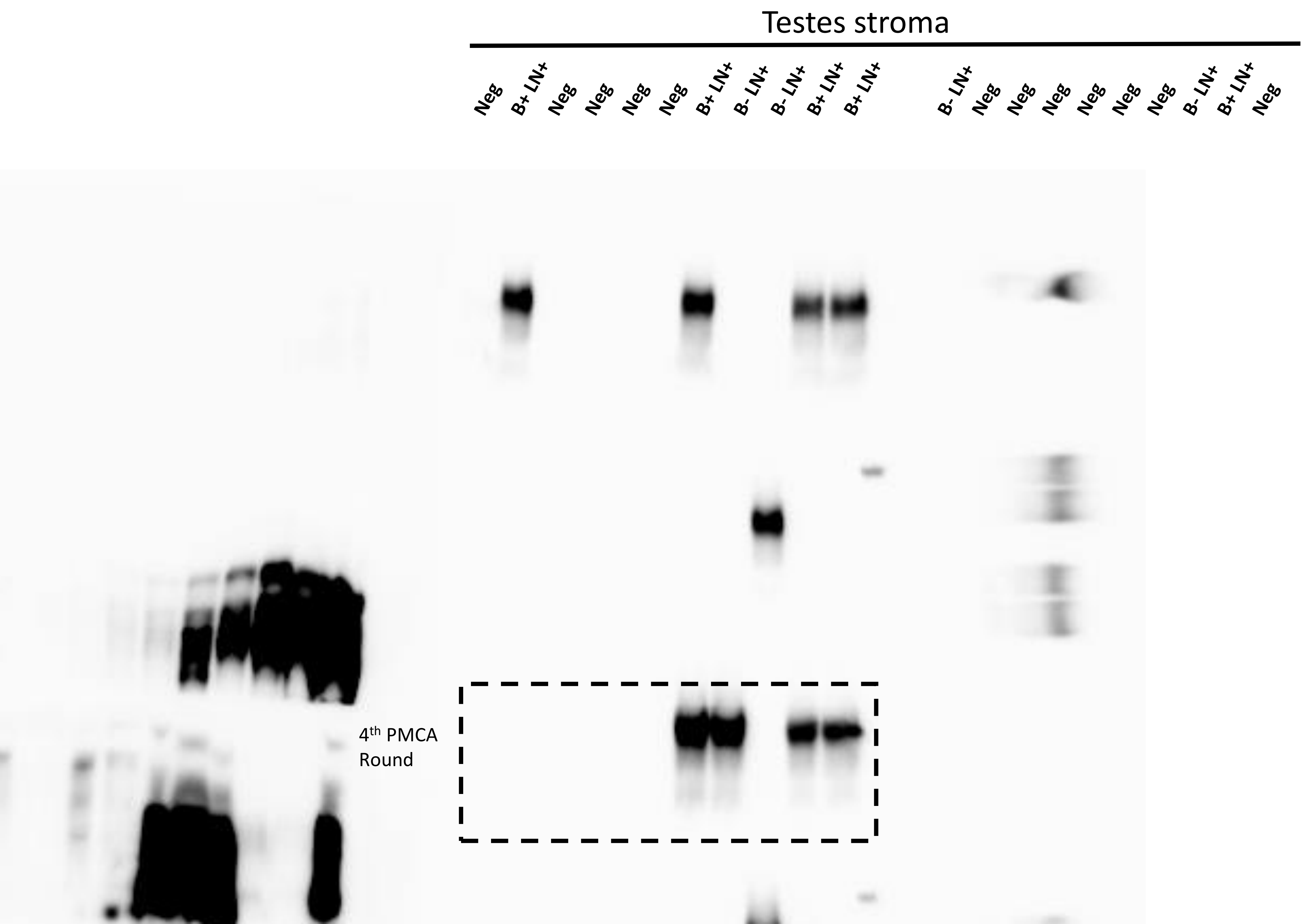
Testes stroma
Neg
B+ LN+
Neg
Neg
Neg
Neg
B+ LN+
B- LN+
B- LN+
B+ LN+
B+ LN+
B- LN+
Neg
Neg
Neg
Neg
Neg
Neg
B- LN+
B+ LN+
Neg
4th PMCA Round

Figure 2

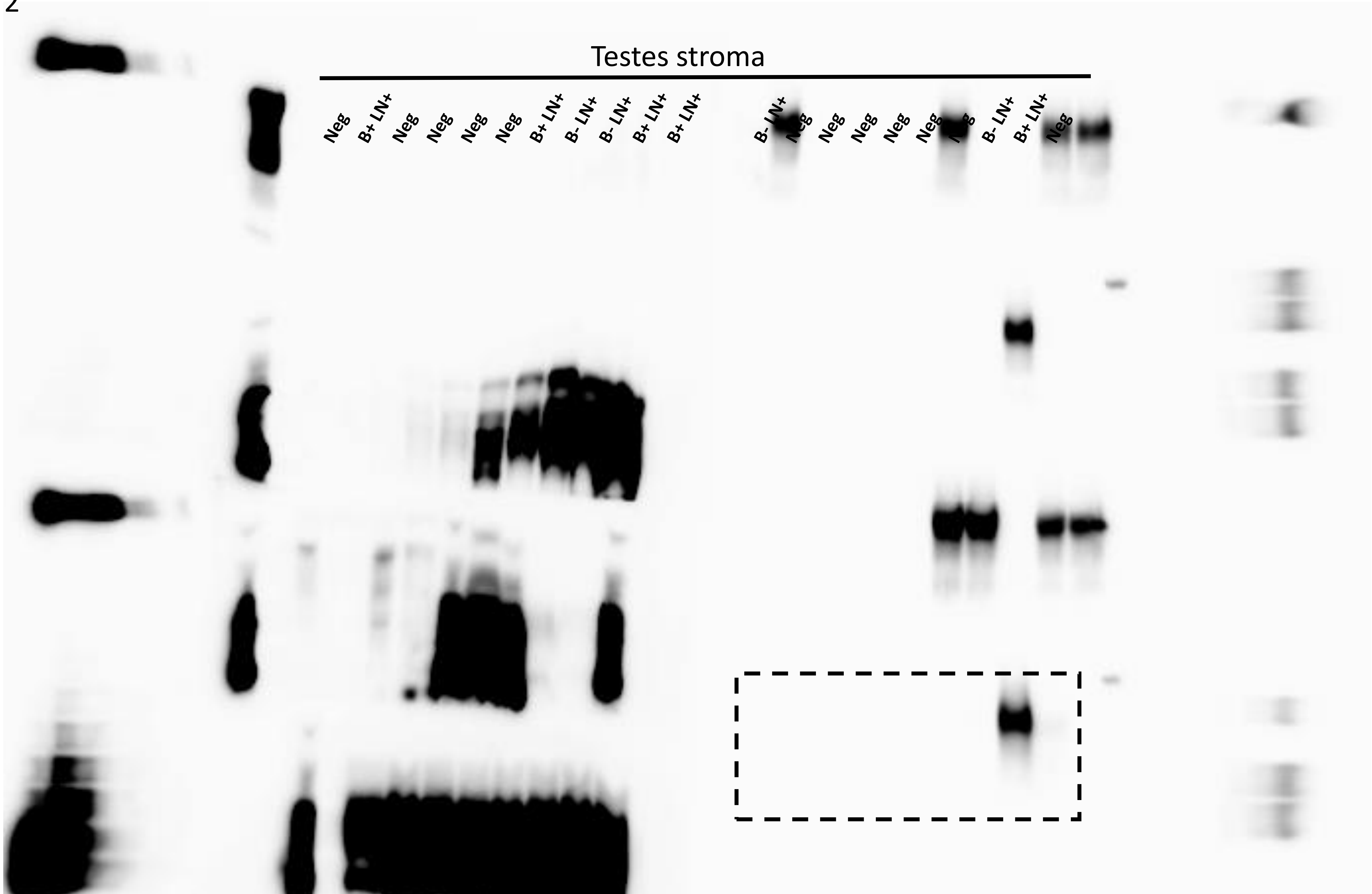
Testes stroma
Neg
B+ LN+
Neg
Neg
Neg
Neg
B+ LN+
B- LN+
B- LN+
B+ LN+
B+ LN+
B- LN+
Neg
Neg
Neg
Neg
Neg
Neg
B- LN+
B+ LN+
Neg

Figure 3

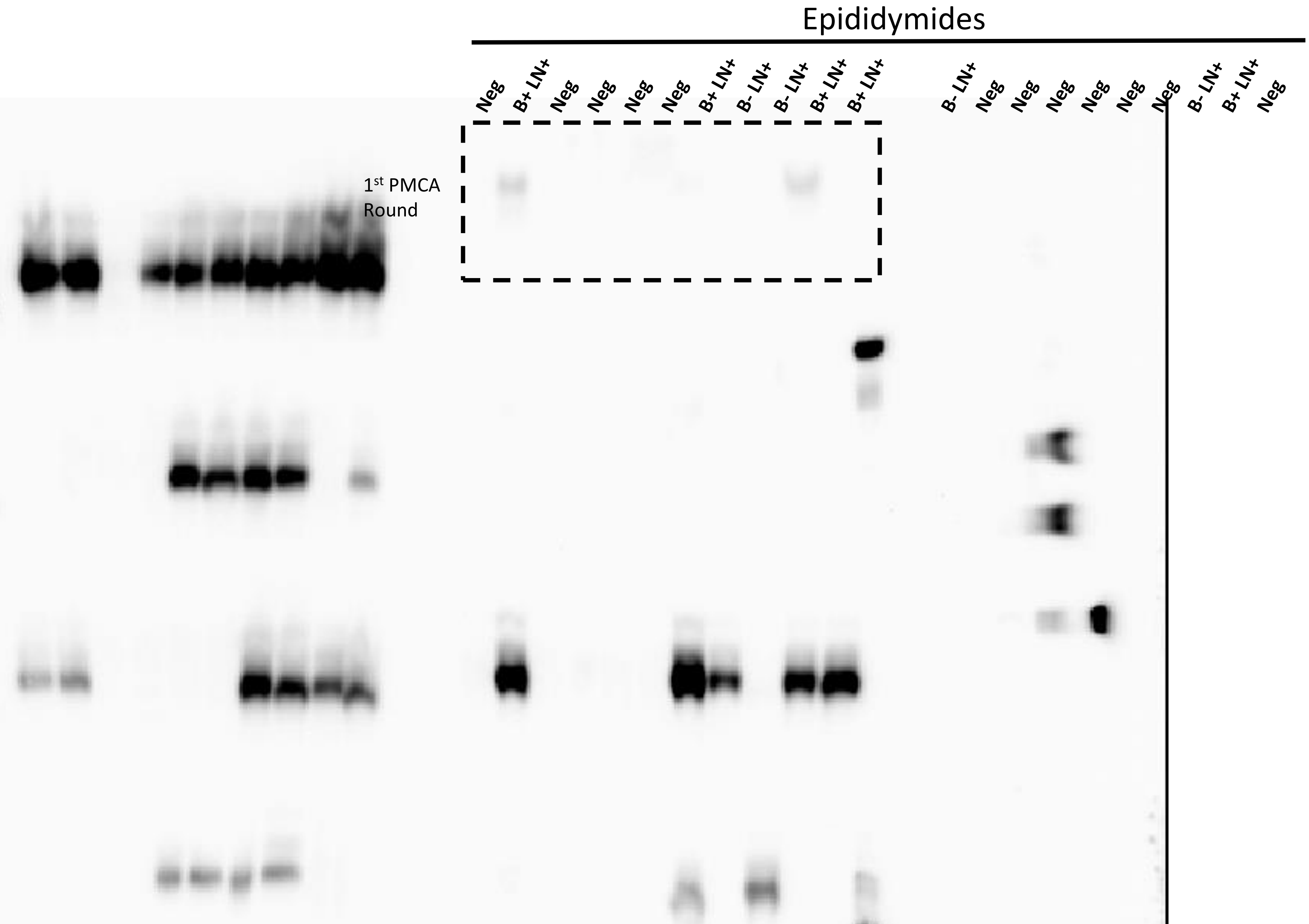
Epididymides
Neg
B+ LN+
Neg
Neg
Neg
Neg
B+ LN+
B- LN+
B- LN+
B+ LN+
B+ LN+
B- LN+
Neg
Neg
Neg
Neg
Neg
Neg
B- LN+
B+ LN+
Neg
1st PMCA Round

Figure 3

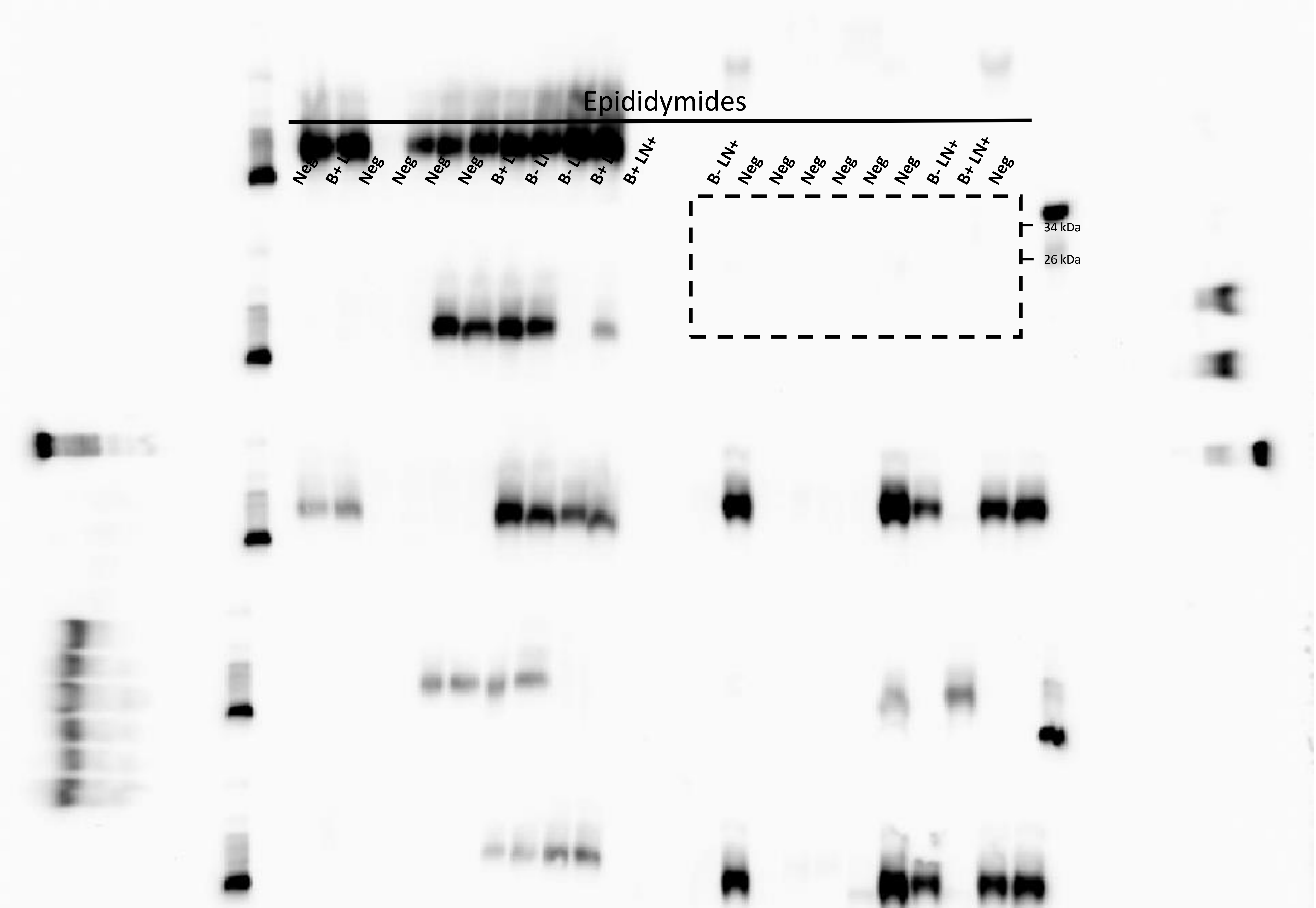
Epididymides
Neg
Neg
Neg
Neg
Neg
B+ LN+
B- LN+
Neg
Neg
Neg
Neg
Neg
Neg
B- LN+
B+ LN+
Neg
34 kDa
26 kDa

Figure 3

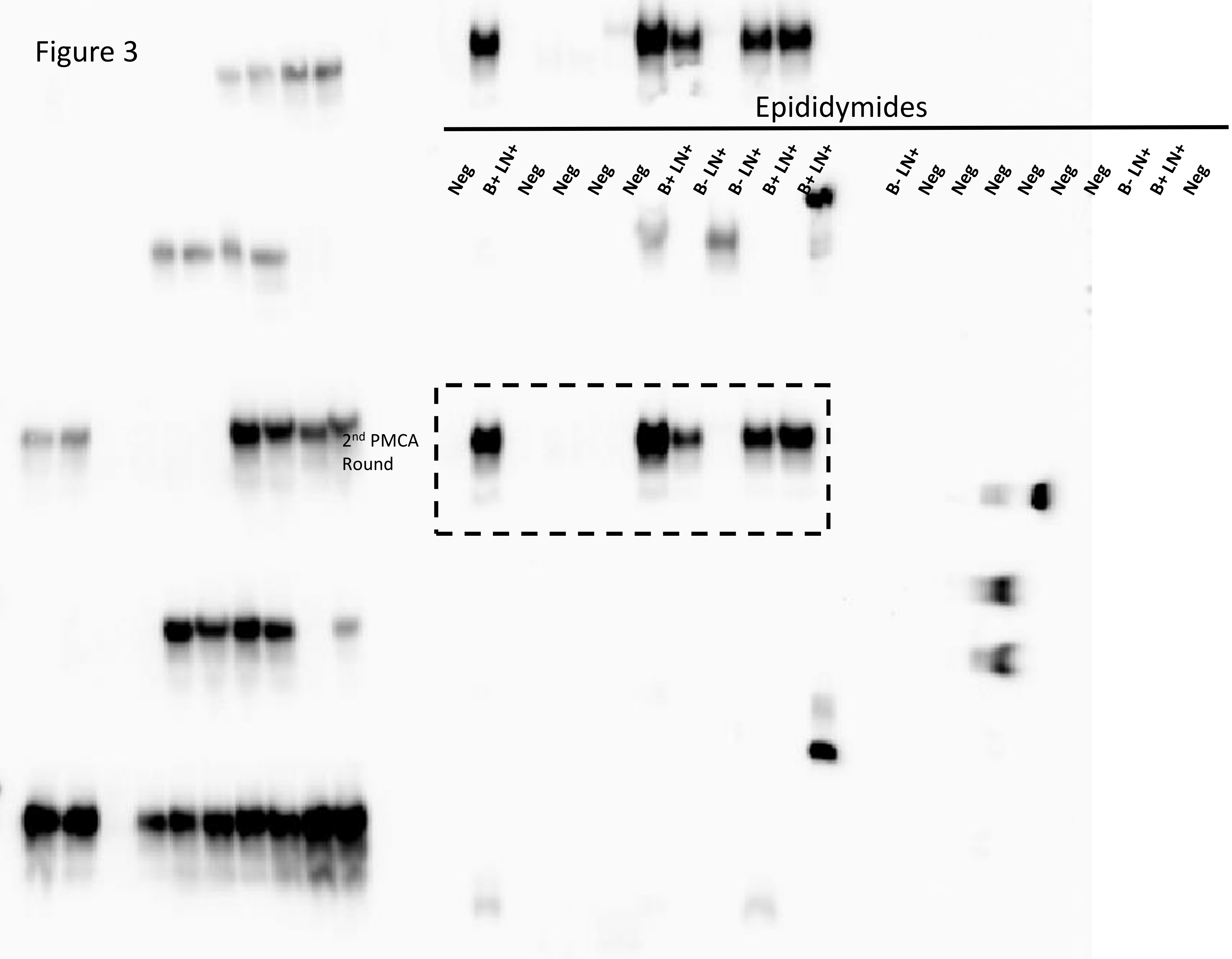

Epididymides
Neg
B+ LN+
Neg
Neg
Neg
Neg
B+ LN+
B- LN+
B- LN+
B+ LN+
B+ LN+
B- LN+
Neg
Neg
Neg
Neg
Neg
Neg
B- LN+
B+ LN+
Neg
2nd PMCA Round

Figure 3

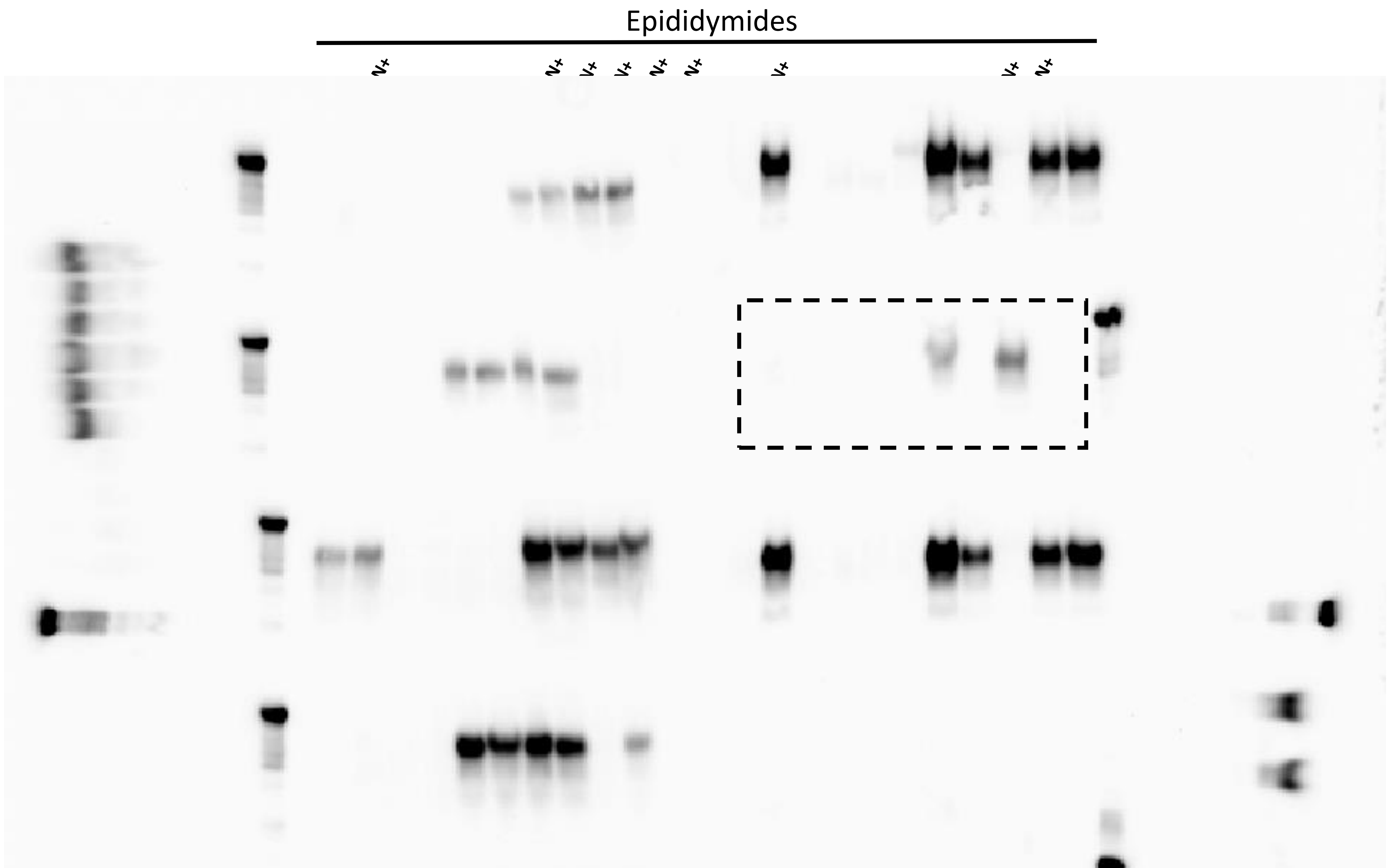

Figure 3

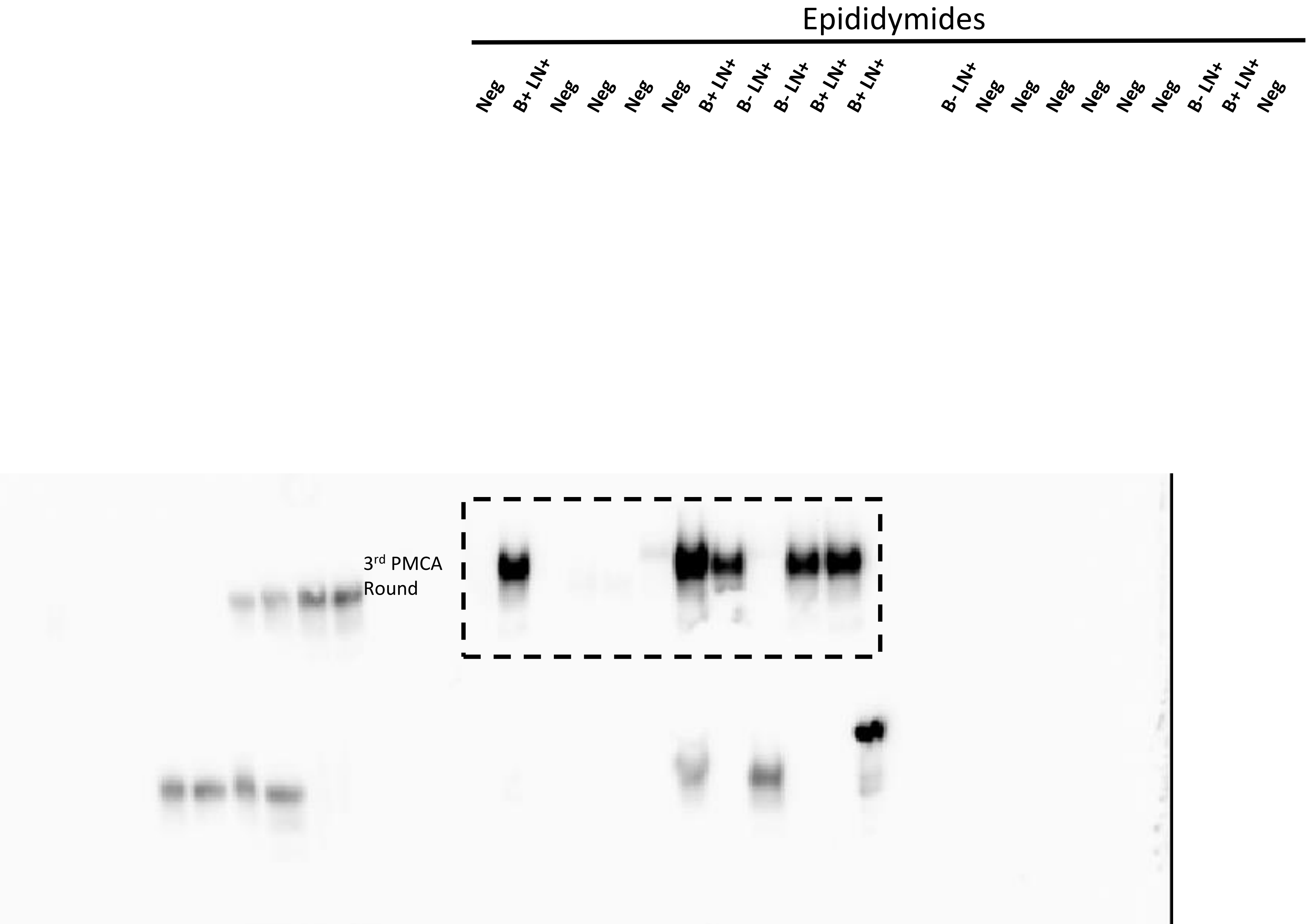
Epididymides
Neg
B+ LN+
Neg
Neg
Neg
Neg
B+ LN+
B- LN+
B- LN+
B+ LN+
B+ LN+
B- LN+
Neg
Neg
Neg
Neg
Neg
Neg
B- LN+
B+ LN+
Neg
3rd PMCA Round

Figure 3

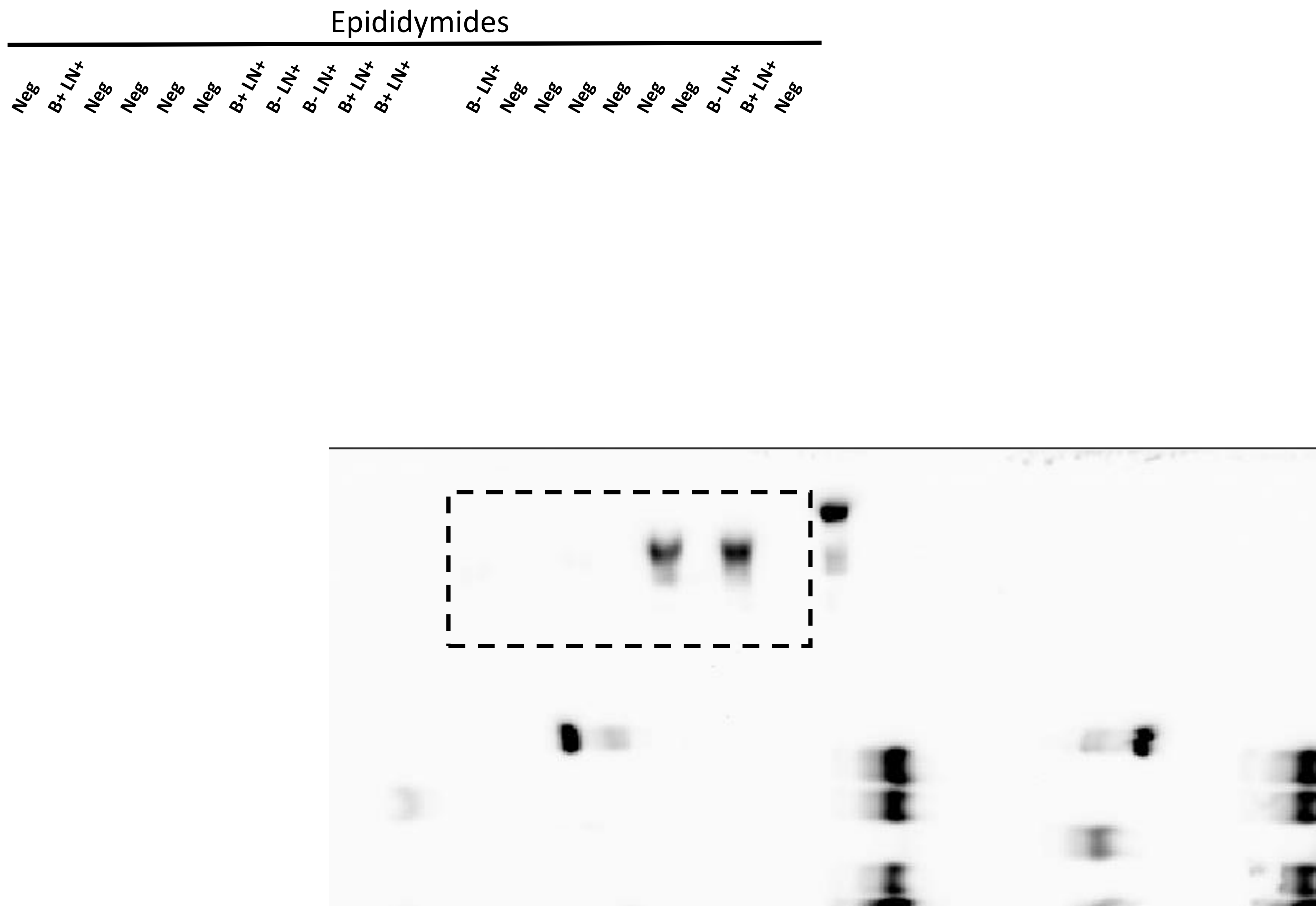
Epididymides
Neg
B+ LN+
Neg
Neg
Neg
Neg
B+ LN+
B- LN+
B- LN+
B+ LN+
B+ LN+
B- LN+
Neg
Neg
Neg
Neg
Neg
Neg
B- LN+
B+ LN+
Neg

Figure 3

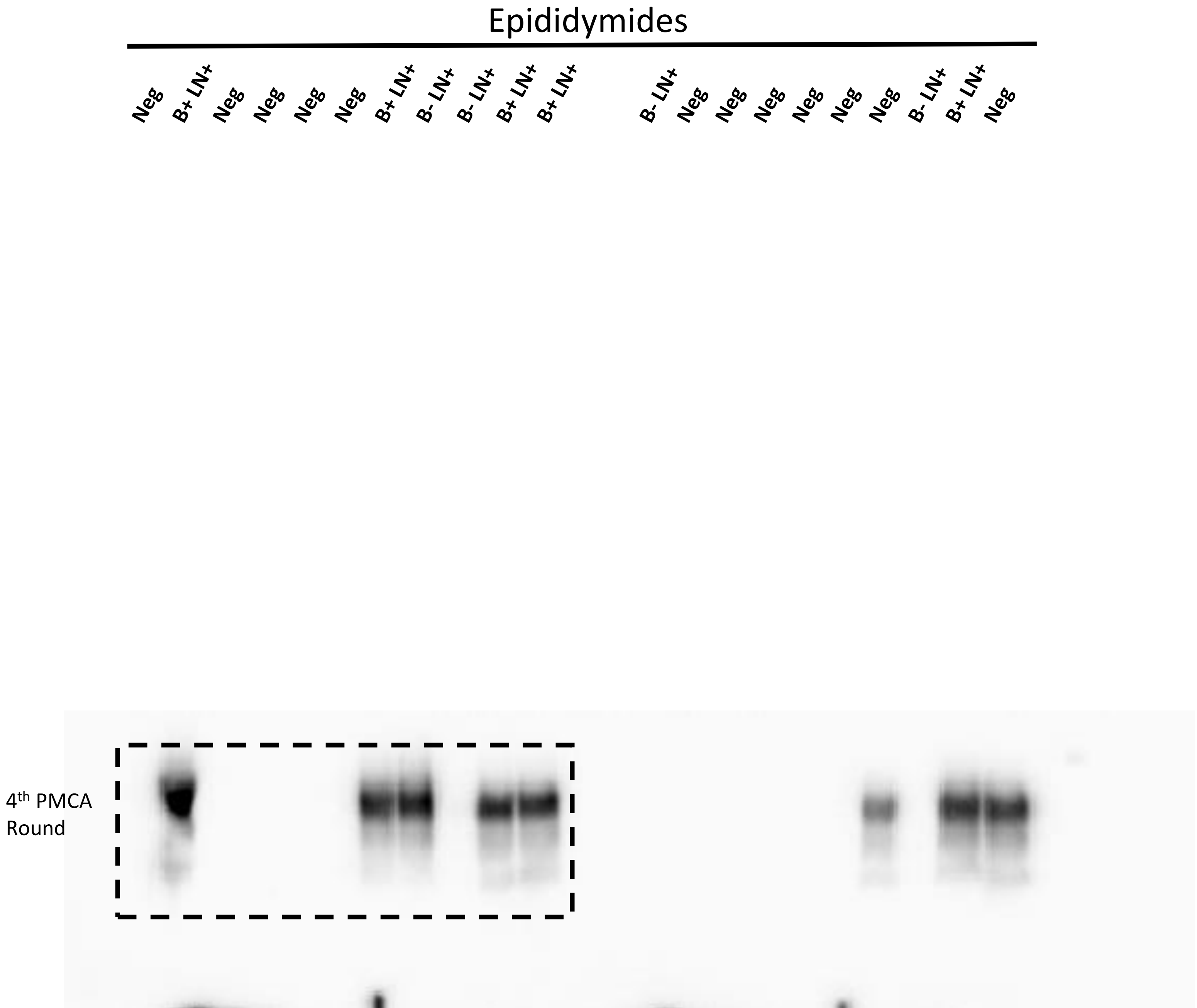
Epididymides
Neg
B+ LN+
Neg
Neg
Neg
Neg
B+ LN+
B- LN+
B- LN+
B+ LN+
B+ LN+
B- LN+
Neg
Neg
Neg
Neg
Neg
Neg
B- LN+
B+ LN+
Neg
4th PMCA Round

Figure 3

Epididymides

Neg B+ LN+ Neg Neg Neg Neg B+ LN+ B- LN+ B- LN+ B+ LN+ B+ LN+ B- LN+ Neg Neg Neg Neg Neg Neg B- LN+ B+ LN+ Neg

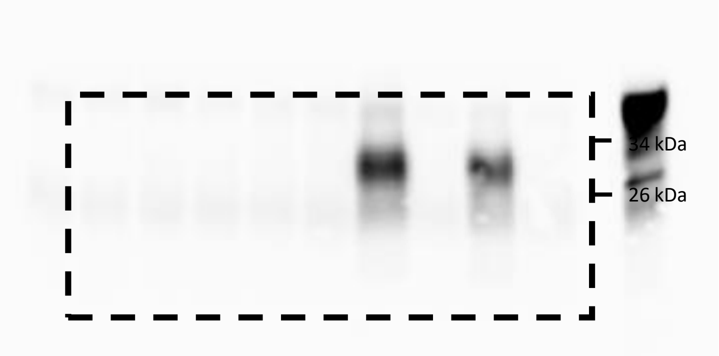

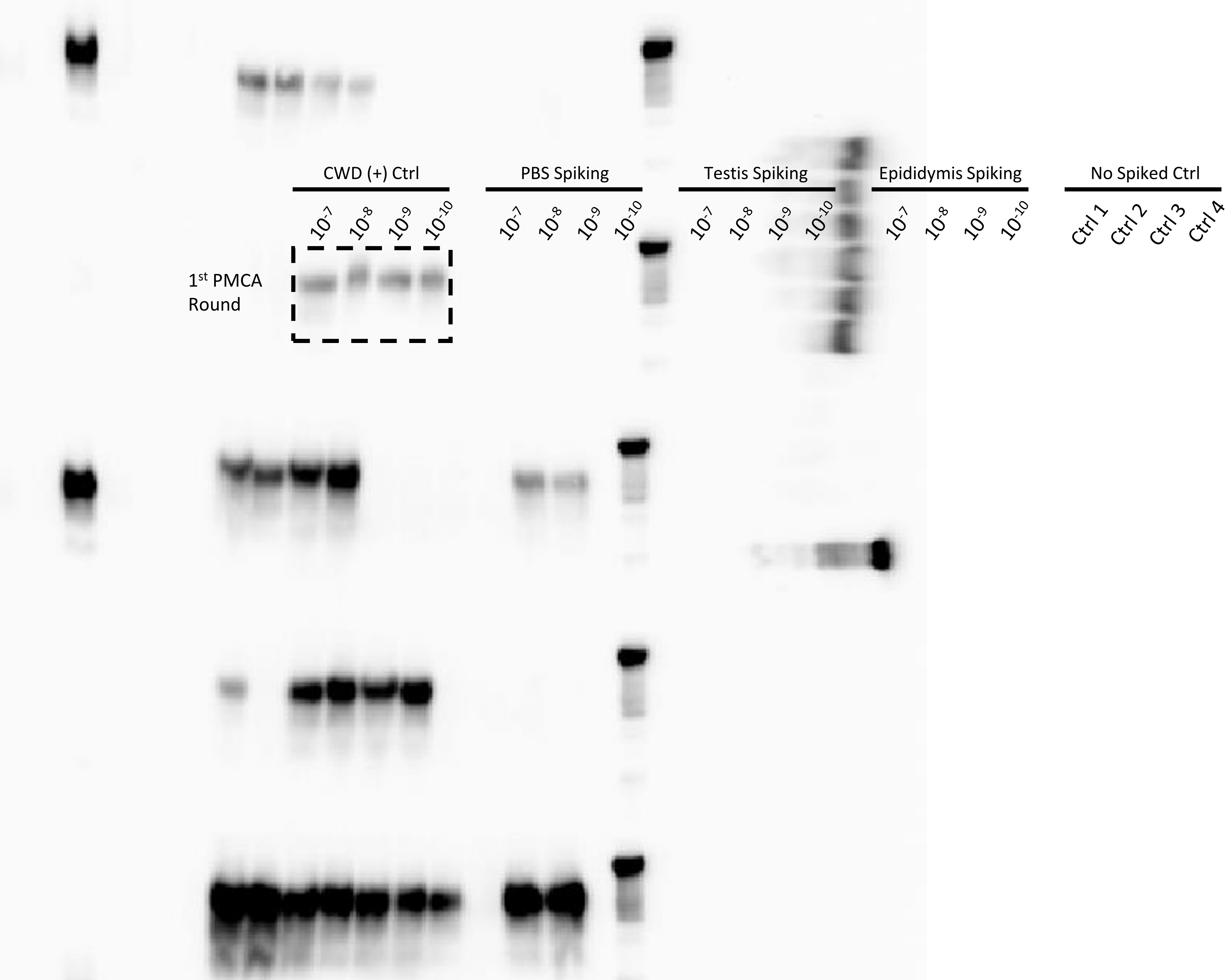
CWD (+) Ctrl
PBS Spiking
Testis Spiking
Epididymis Spiking
No Spiked Ctrl
10-7
10-8
10-9
10-10
Ctrl 1
Ctrl 2
Ctrl 3
Ctrl 4
1st PMCA Round

Figure 4

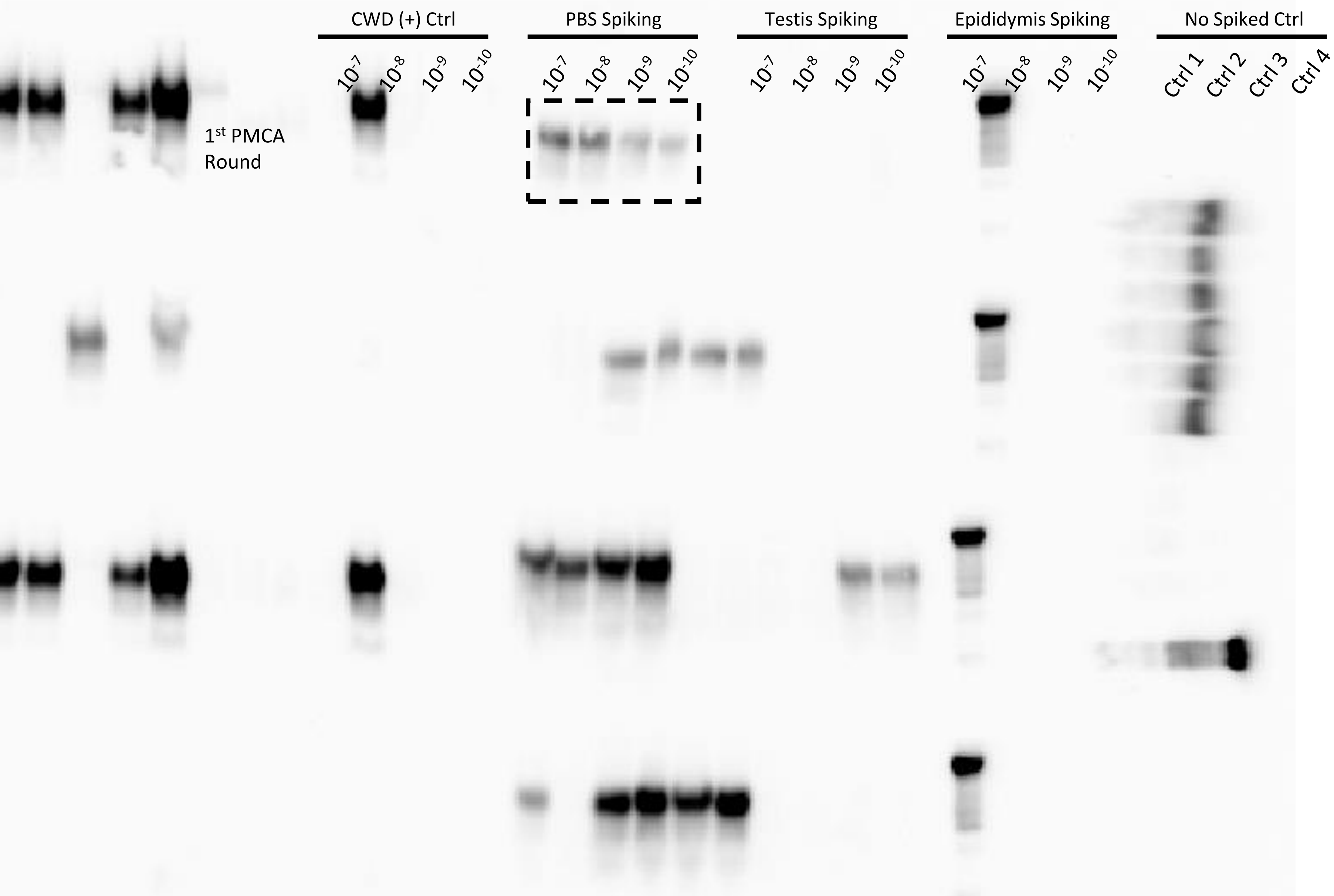
CWD (+) Ctrl
PBS Spiking
Testis Spiking
Epididymis Spiking
No Spiked Ctrl
10-7
10-8
10-9
10-10
Ctrl 1
Ctrl 2
Ctrl 3
Ctrl 4
1st PMCA Round

Figure 4

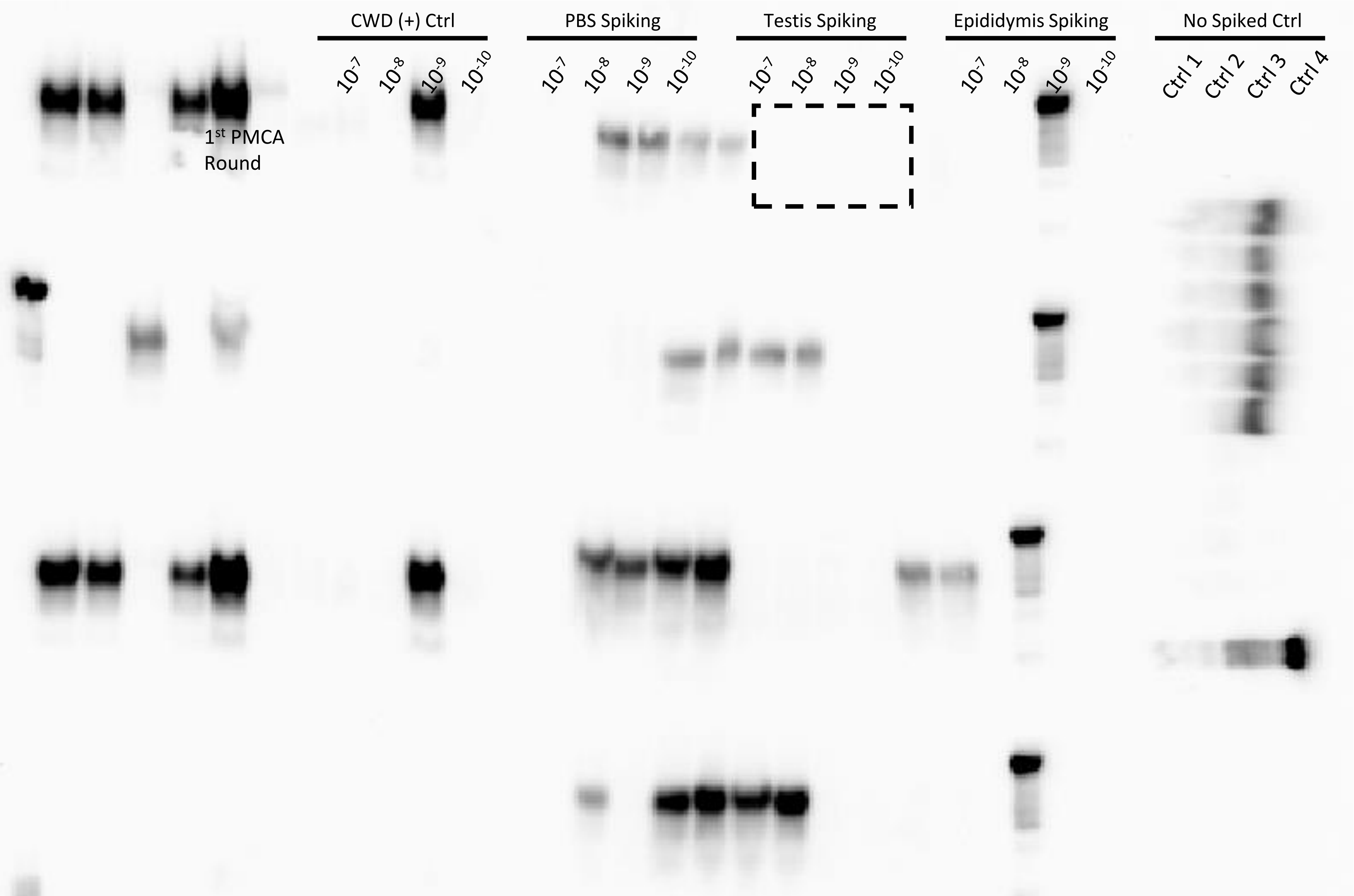

CWD (+) Ctrl
PBS Spiking
Testis Spiking
Epididymis Spiking
No Spiked Ctrl
10-7
10-8
10-9
10-10
10-7
10-8
10-9
10-10
10-7
10-8
10-9
10-10
10-7
10-8
10-9
10-10
Ctrl 1
Ctrl 2
Ctrl 3
Ctrl 4
1st PMCA Round

Figure 4

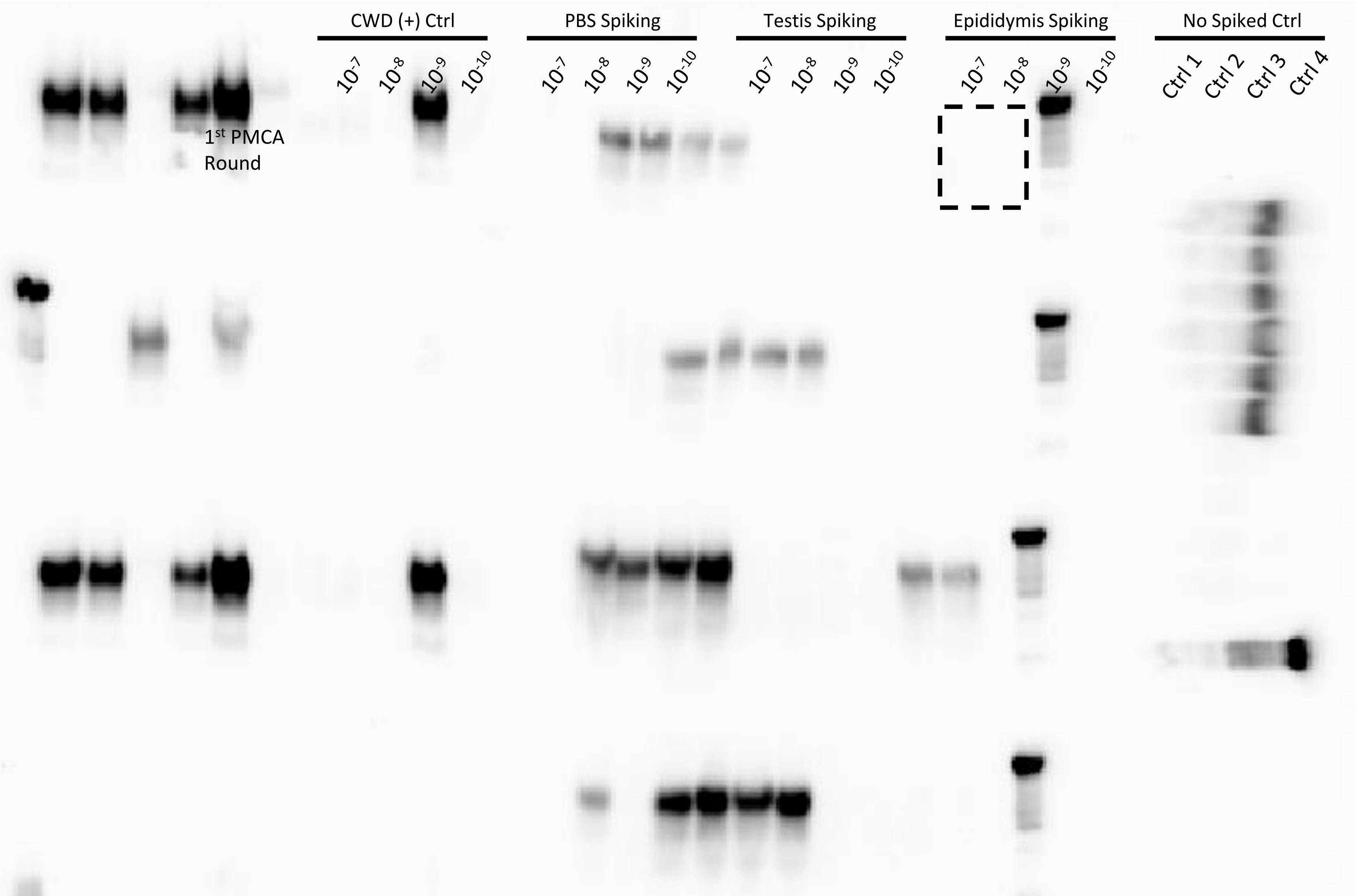

CWD (+) Ctrl
PBS Spiking
Testis Spiking
Epididymis Spiking
No Spiked Ctrl
10-7
10-8
10-9
10-10
10-7
10-8
10-9
10-10
10-7
10-8
10-9
10-10
10-7
10-8
10-9
10-10
Ctrl 1
Ctrl 2
Ctrl 3
Ctrl 4
1st PMCA Round

Figure 4

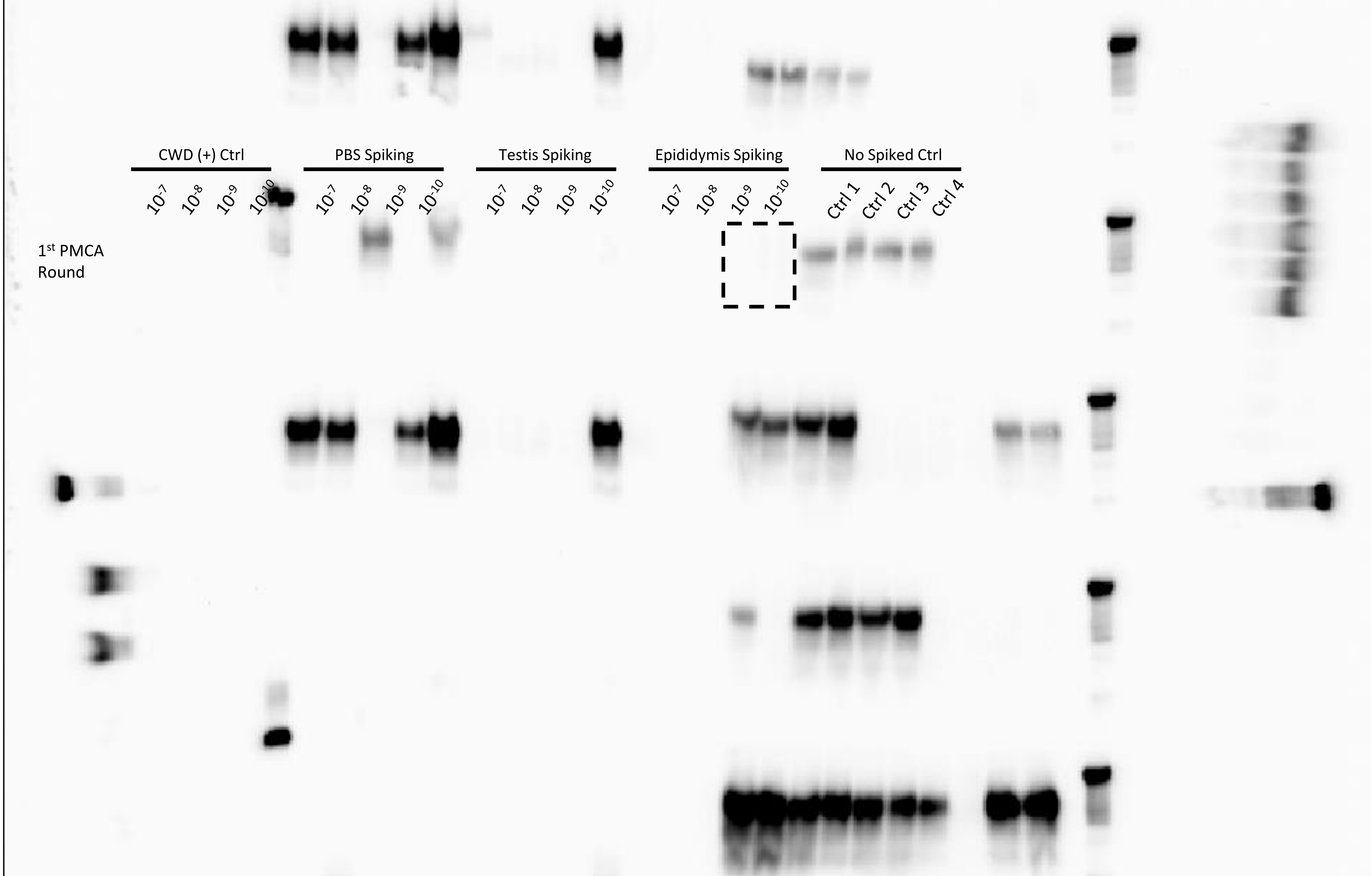

CWD (+) Ctrl
PBS Spiking
Testis Spiking
Epididymis Spiking
No Spiked Ctrl
10-7
10-8
10-9
10-10
10-7
10-8
10-9
10-10
10-7
10-8
10-9
10-10
10-7
10-8
10-9
10-10
Ctrl 1
Ctrl 2
Ctrl 3
Ctrl 4
1st PMCA Round

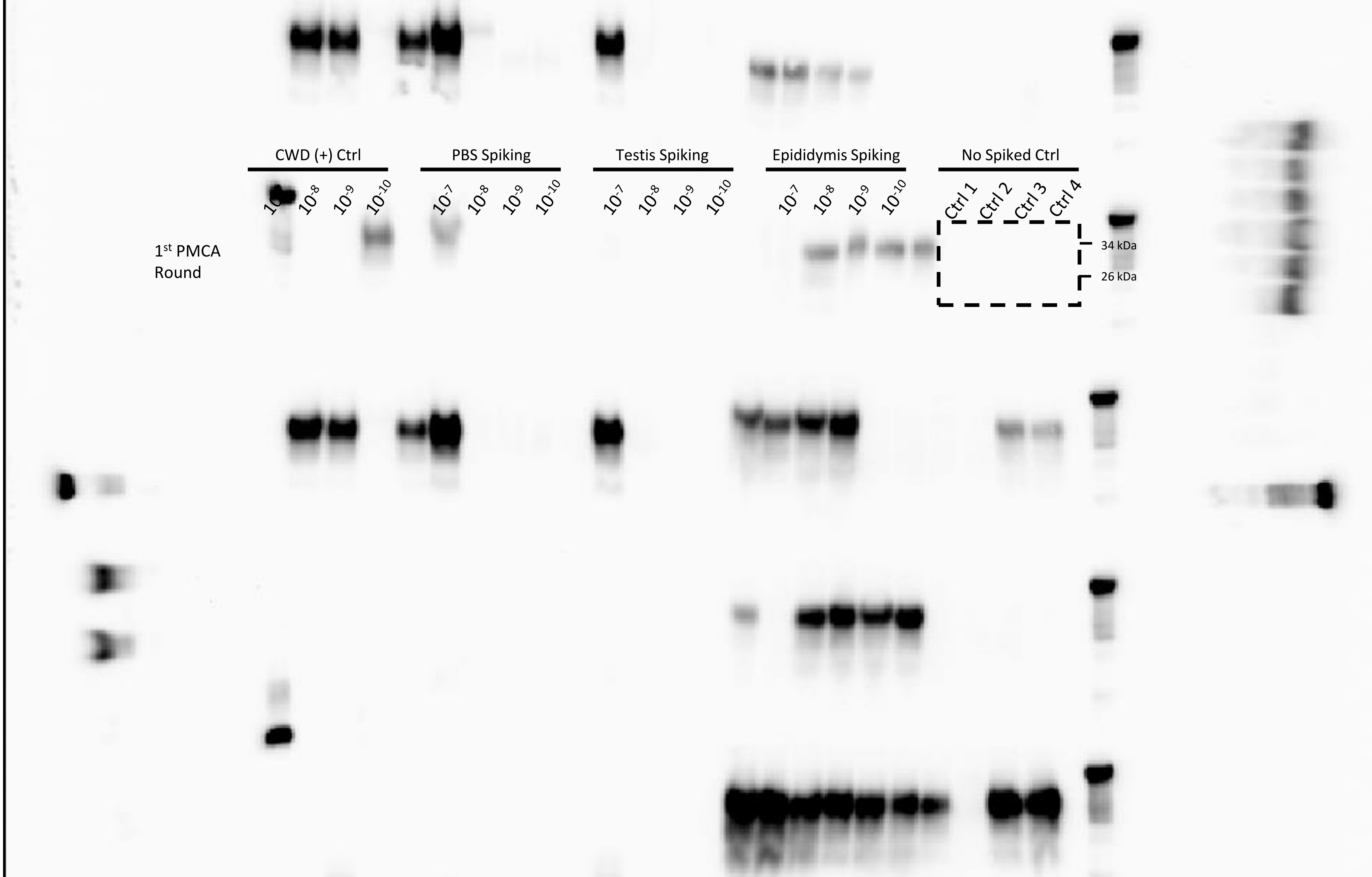
CWD (+) Ctrl
PBS Spiking
Testis Spiking
Epididymis Spiking
No Spiked Ctrl
10-7
10-8
10-9
10-10
Ctrl 1
Ctrl 2
Ctrl 3
Ctrl 4
1st PMCA Round
34 kDa
26 kDa

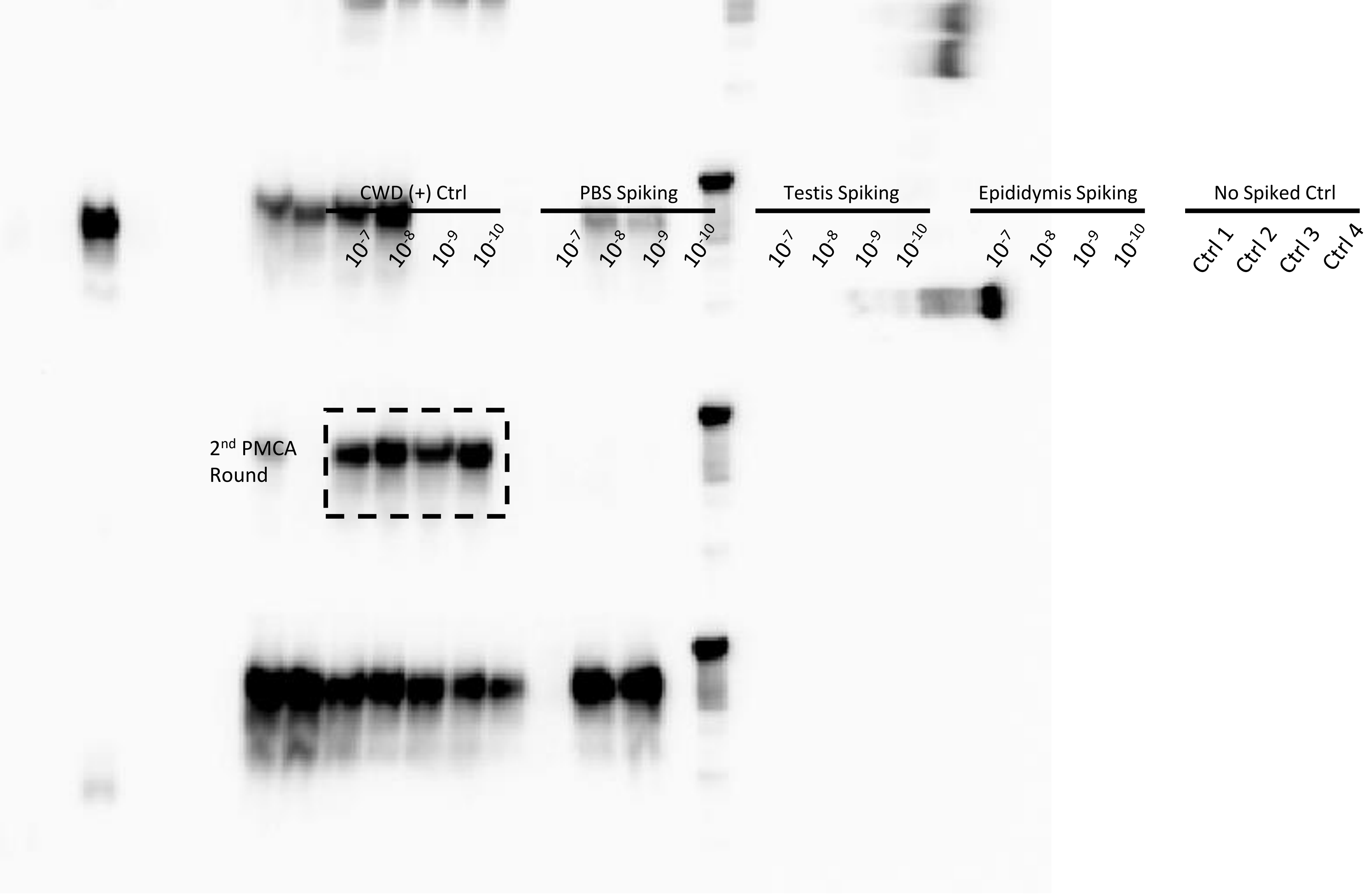

CWD (+) Ctrl
PBS Spiking
Testis Spiking
Epididymis Spiking
No Spiked Ctrl
10-7
10-8
10-9
10-10
10-7
10-8
10-9
10-10
10-7
10-8
10-9
10-10
10-7
10-8
10-9
10-10
Ctrl 1
Ctrl 2
Ctrl 3
Ctrl 4
2nd PMCA
Round

Figure 4

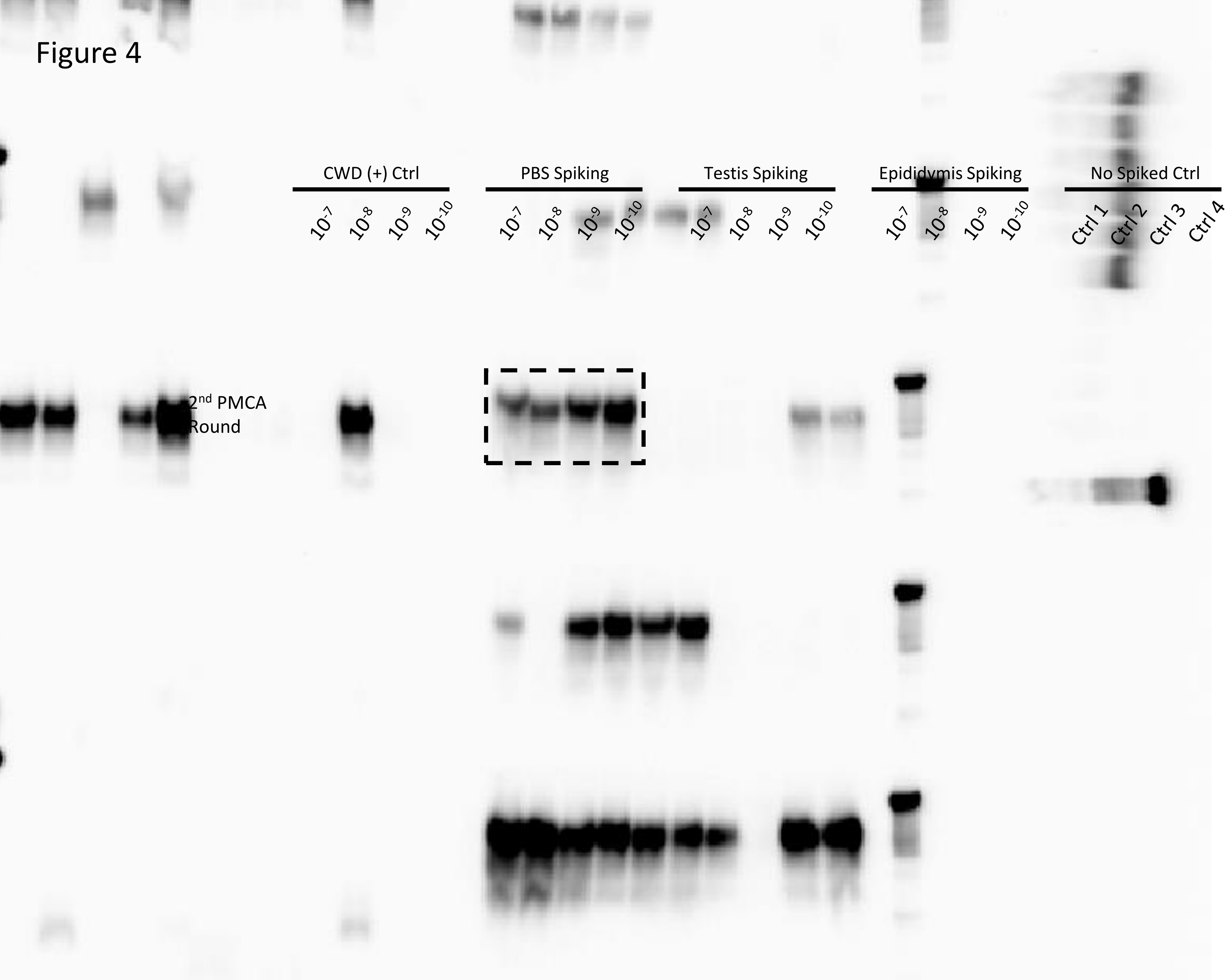

CWD (+) Ctrl
PBS Spiking
Testis Spiking
Epididymis Spiking
No Spiked Ctrl
10-7
10-8
10-9
10-10
10-7
10-8
10-9
10-10
10-7
10-8
10-9
10-10
10-7
10-8
10-9
10-10
Ctrl 1
Ctrl 2
Ctrl 3
Ctrl 4
2nd PMCA Round

Figure 4

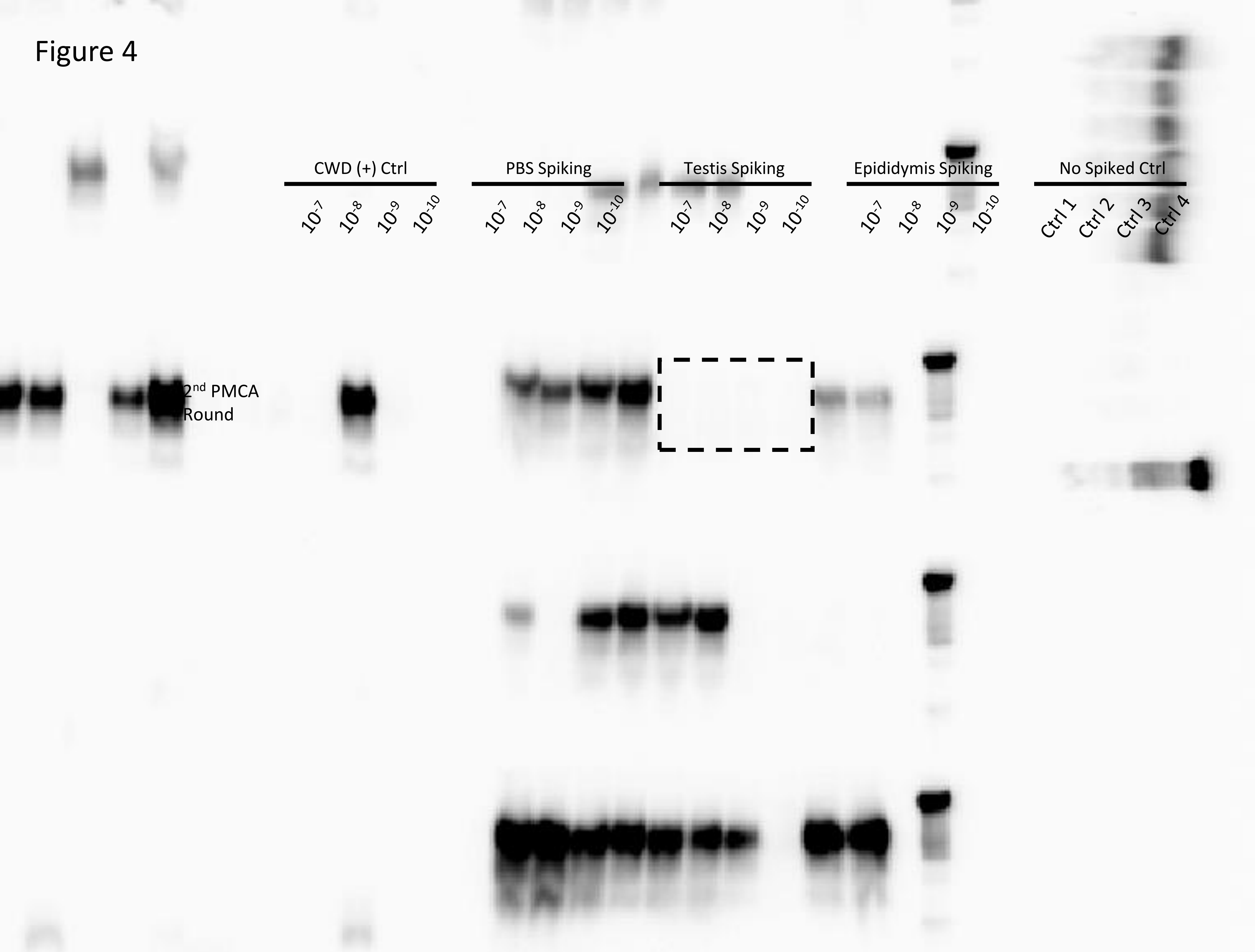

CWD (+) Ctrl
PBS Spiking
Testis Spiking
Epididymis Spiking
No Spiked Ctrl
10-7
10-8
10-9
10-10
Ctrl 1
Ctrl 2
Ctrl 3
Ctrl 4
2nd PMCA Round

Figure 4

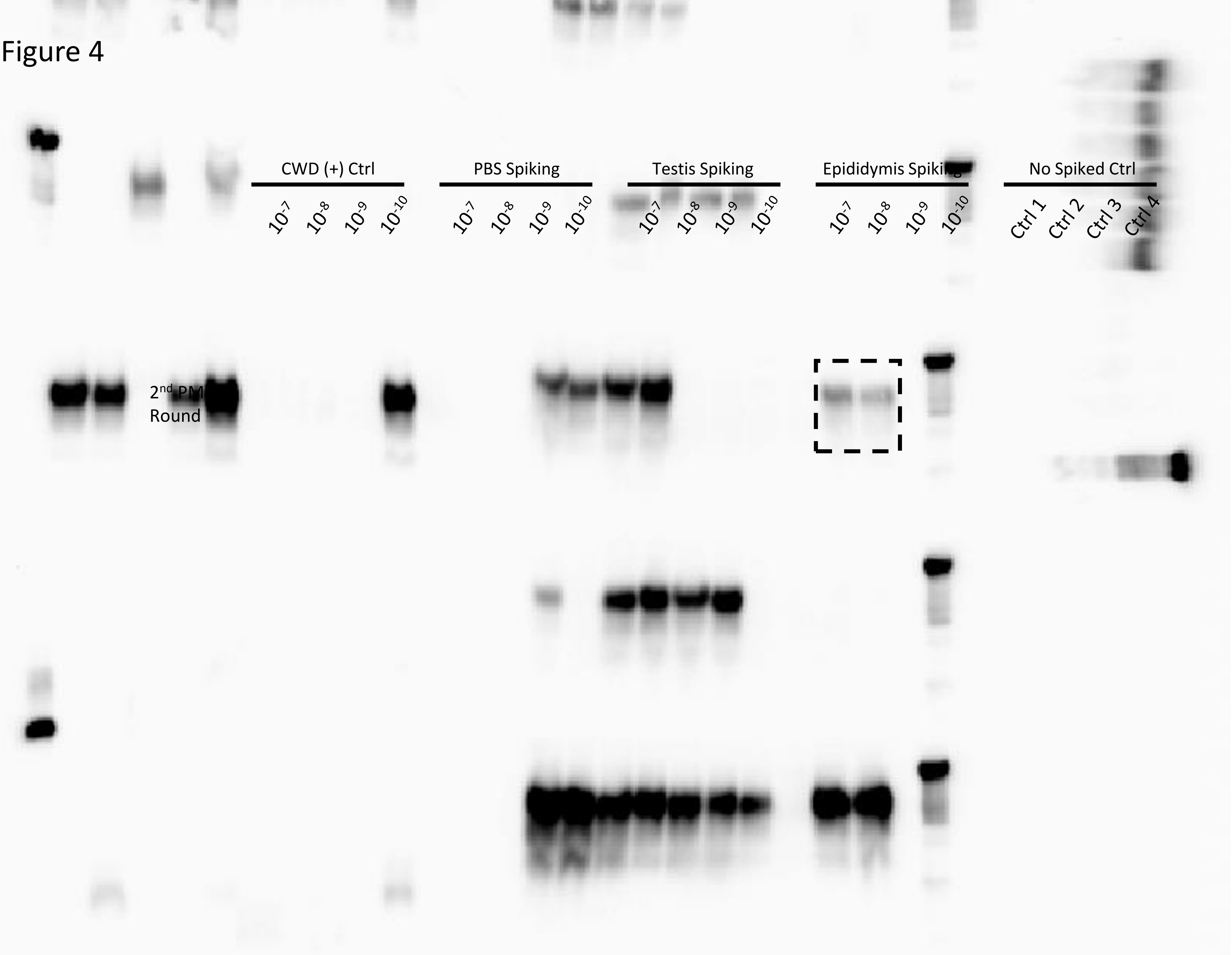
CWD (+) Ctrl
PBS Spiking
Testis Spiking
Epididymis Spiking
No Spiked Ctrl
10^{-7} 10^{-8} 10^{-9} 10^{-10}
10^{-7} 10^{-8} 10^{-9} 10^{-10}
10^{-7} 10^{-8} 10^{-9} 10^{-10}
10^{-7} 10^{-8} 10^{-9} 10^{-10}
Ctrl 1
Ctrl 2
Ctrl 3
Ctrl 4
2nd PM
Round

Figure 4

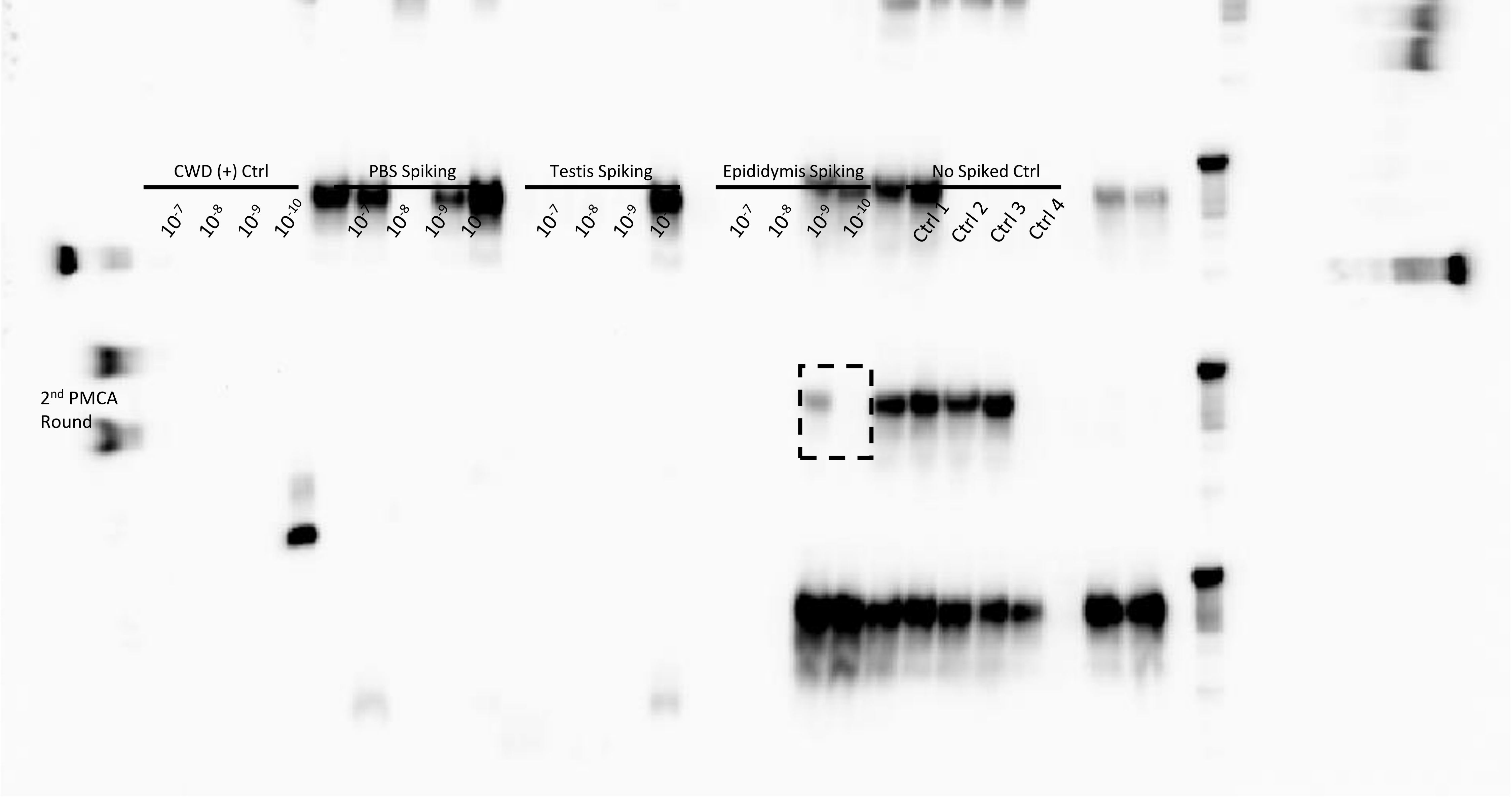
CWD (+) Ctrl
PBS Spiking
Testis Spiking
Epididymis Spiking
No Spiked Ctrl
10-7
10-8
10-9
10-10
Ctrl 1
Ctrl 2
Ctrl 3
Ctrl 4
2nd PMCA Round

Figure 4

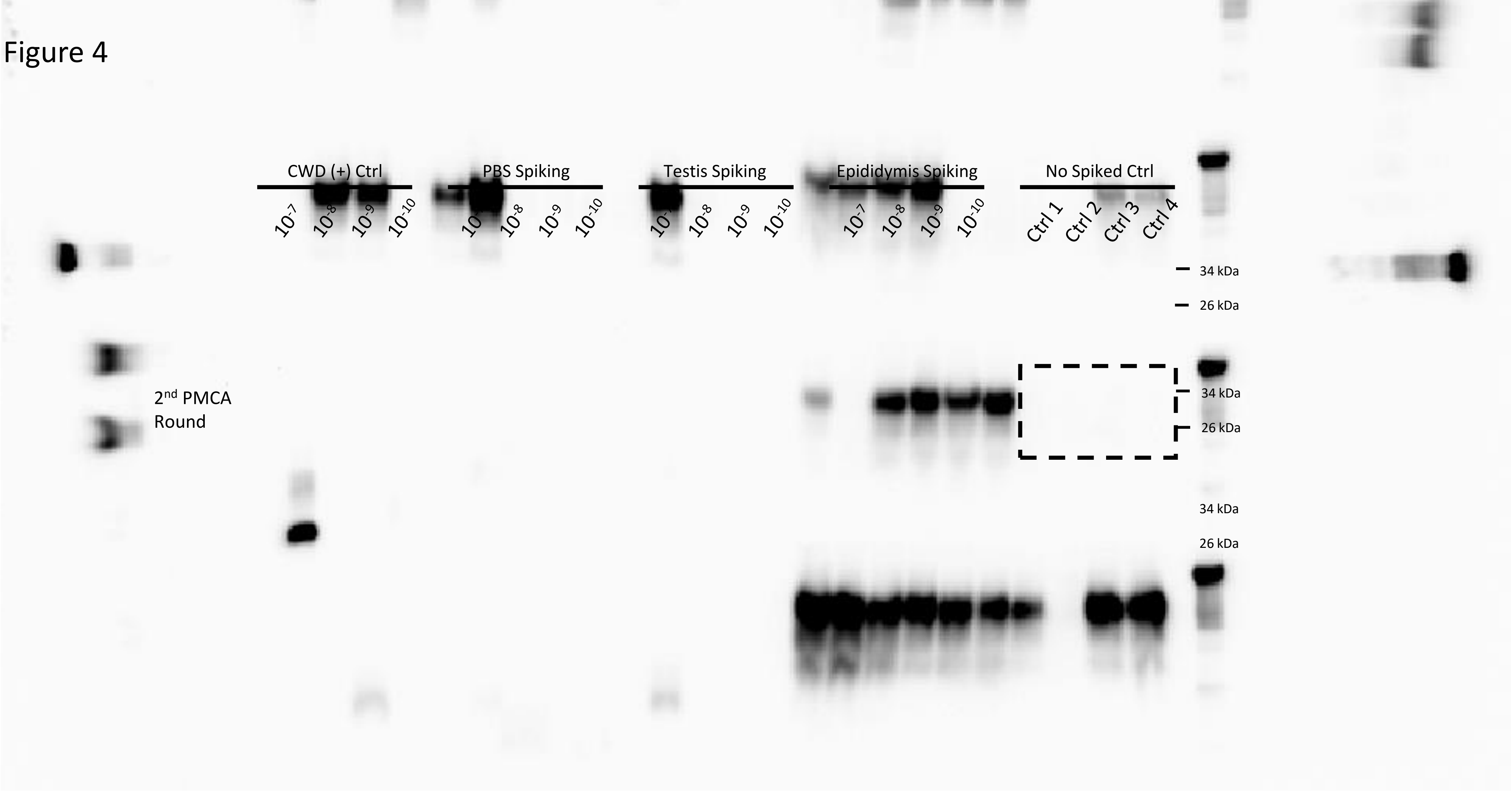

CWD (+) Ctrl
PBS Spiking
Testis Spiking
Epididymis Spiking
No Spiked Ctrl
10-7 10-8 10-9 10-10
Ctrl 1 Ctrl 2 Ctrl 3 Ctrl 4
34 kDa
26 kDa
2nd PMCA Round

Figure 4

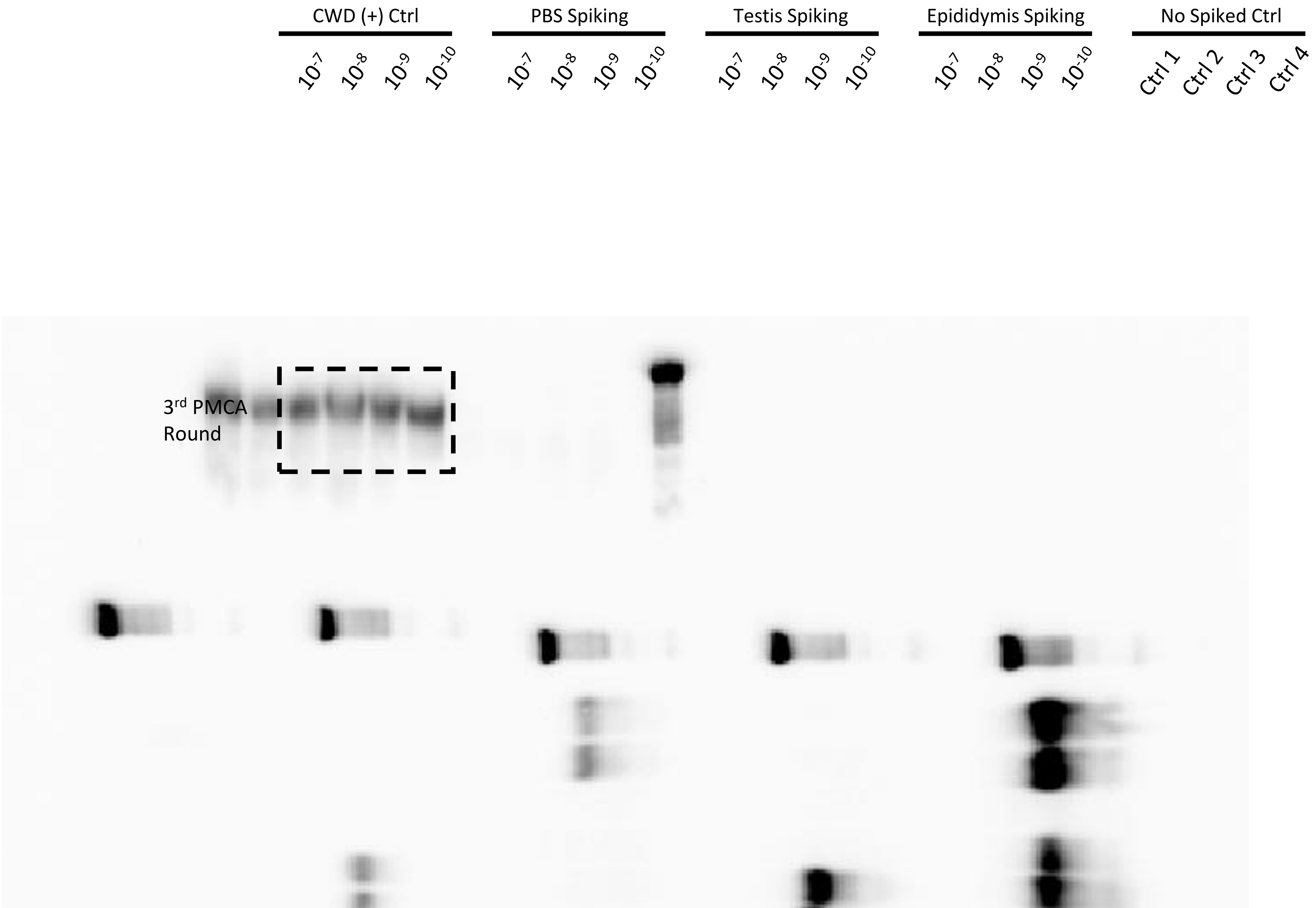
CWD (+) Ctrl
PBS Spiking
Testis Spiking
Epididymis Spiking
No Spiked Ctrl
10-7
10-8
10-9
10-10
Ctrl 1
Ctrl 2
Ctrl 3
Ctrl 4
3rd PMCA Round

Figure 4

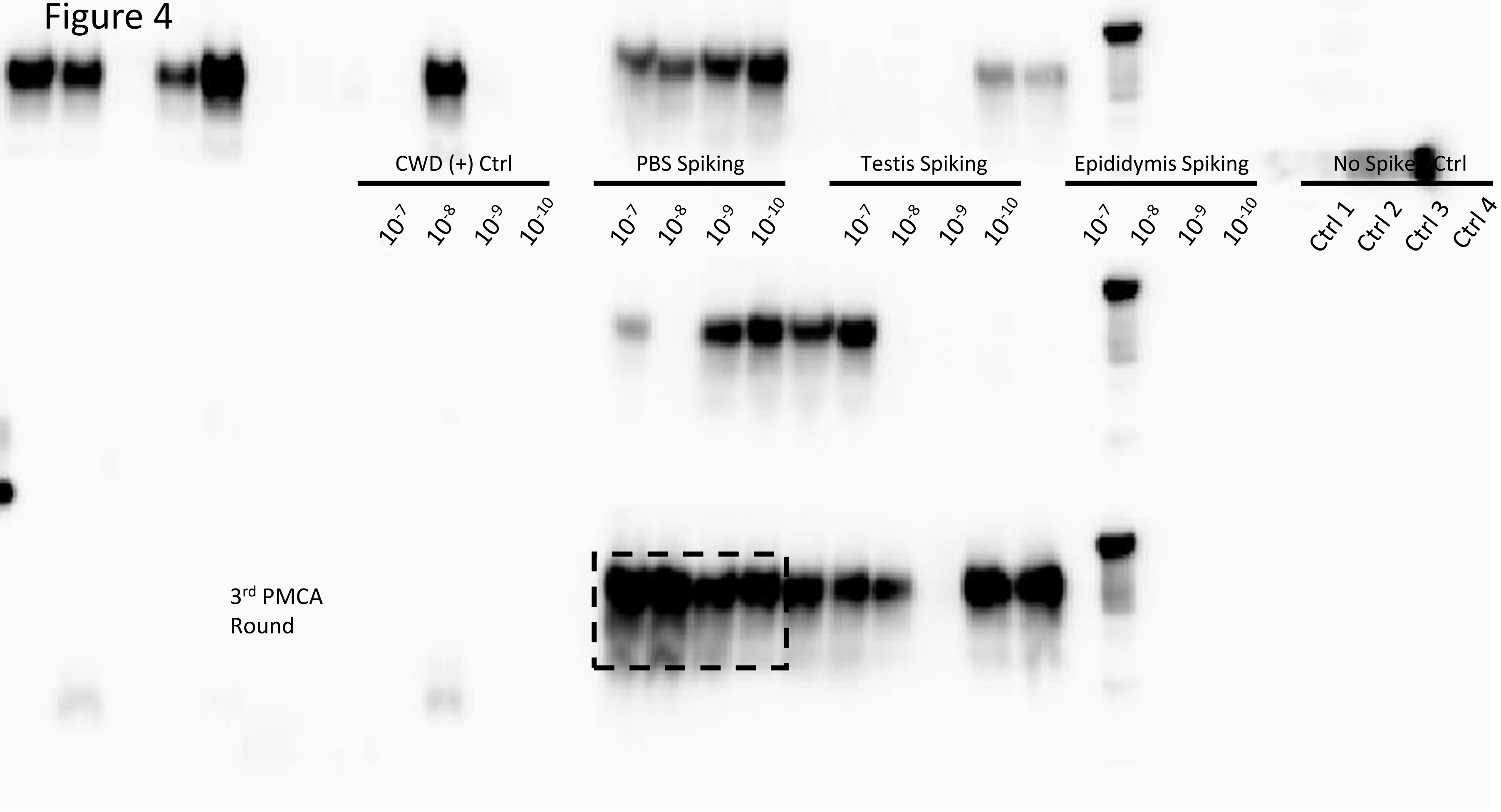
CWD (+) Ctrl
PBS Spiking
Testis Spiking
Epididymis Spiking
No Spike Ctrl
10-7
10-8
10-9
10-10
Ctrl 1
Ctrl 2
Ctrl 3
Ctrl 4
3rd PMCA Round

Figure 4

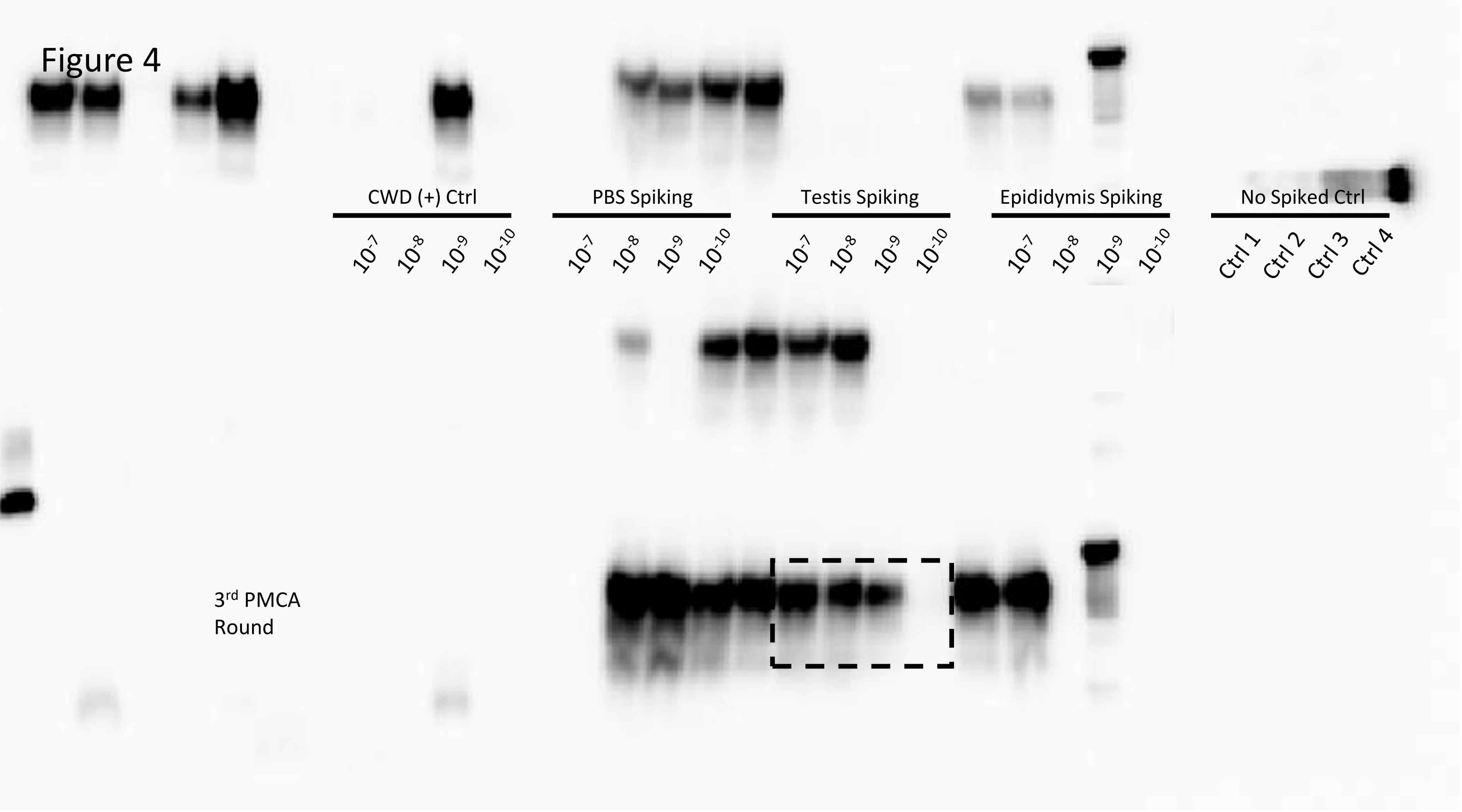
CWD (+) Ctrl
PBS Spiking
Testis Spiking
Epididymis Spiking
No Spiked Ctrl
10-7
10-8
10-9
10-10
Ctrl 1
Ctrl 2
Ctrl 3
Ctrl 4
3rd PMCA Round

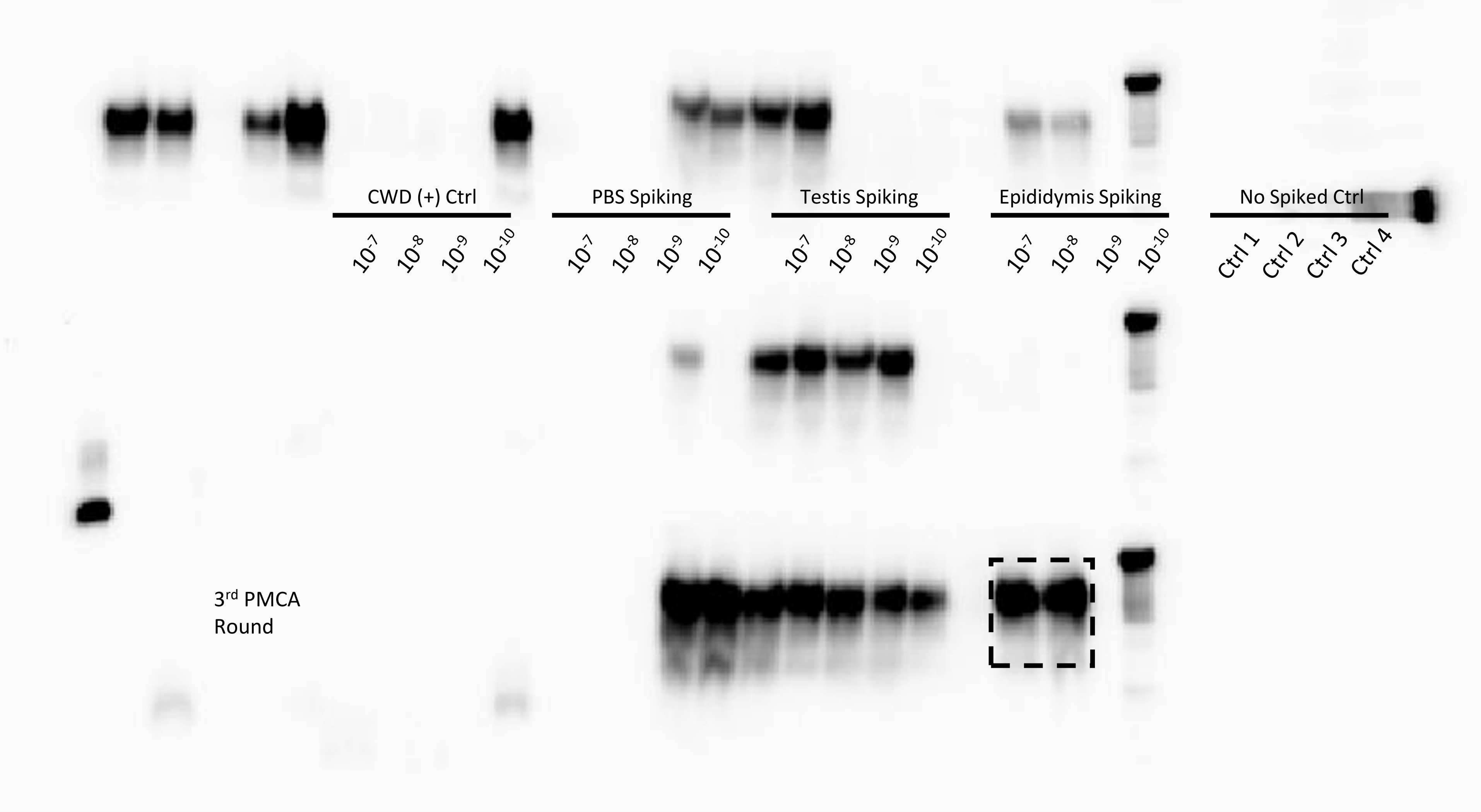
CWD (+) Ctrl
PBS Spiking
Testis Spiking
Epididymis Spiking
No Spiked Ctrl
10-7
10-8
10-9
10-10
Ctrl 1
Ctrl 2
Ctrl 3
Ctrl 4
3rd PMCA Round

Figure 4

CWD (+) Ctrl				PBS Spiking				Testis Spiking				Epididymis Spiking				No Spiked Ctrl			
10^{-7}	10^{-8}	10^{-9}	10^{-10}	10^{-7}	10^{-8}	10^{-9}	10^{-10}	10^{-7}	10^{-8}	10^{-9}	10^{-10}	10^{-7}	10^{-8}	10^{-9}	10^{-10}	Ctrl 1	Ctrl 2	Ctrl 3	Ctrl 4

3rd PMCA Round

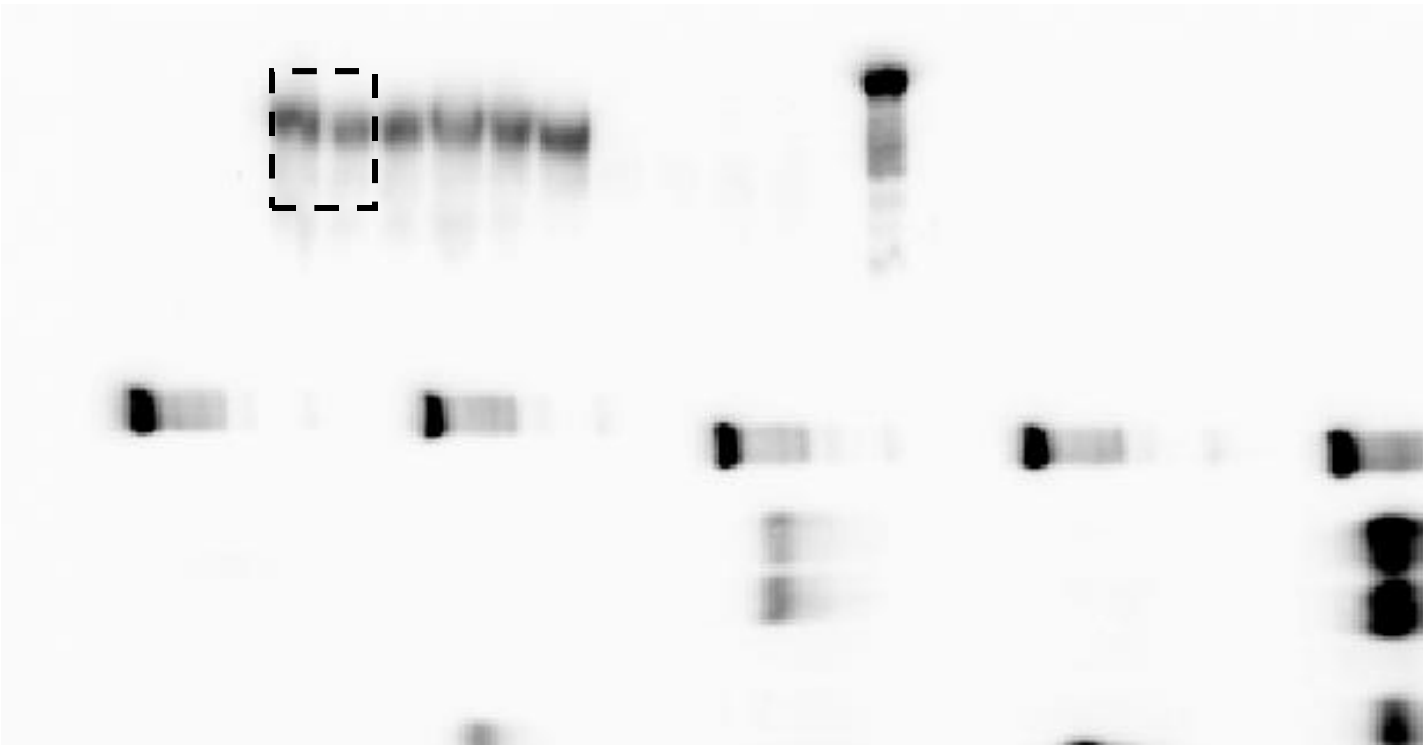

Figure 4

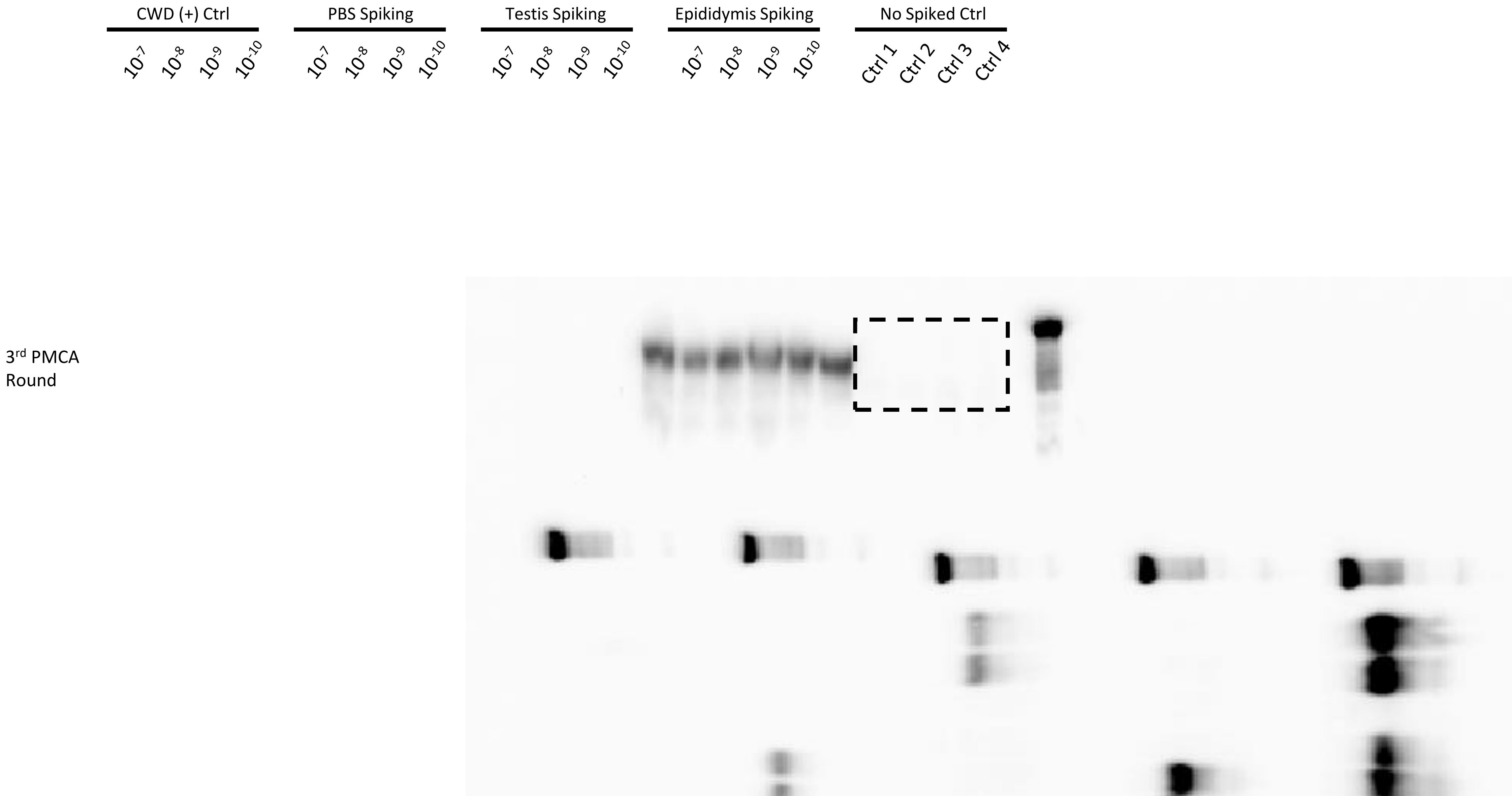
CWD (+) Ctrl
PBS Spiking
Testis Spiking
Epididymis Spiking
No Spiked Ctrl
10-7
10-8
10-9
10-10
10-7
10-8
10-9
10-10
10-7
10-8
10-9
10-10
10-7
10-8
10-9
10-10
Ctrl 1
Ctrl 2
Ctrl 3
Ctrl 4
3rd PMCA Round
