# Supplementary material for: In Vitro detection of Chronic Wasting Disease (CWD) prions in semen and reproductive tissues of white tailed deer bucks (Odocoileus virginianus)
Source: PLoS One. 2019 Dec 30;14(12):e0226560. doi: 10.1371/journal.pone.0226560 (PMC6936793; doi:10.1371/journal.pone.0226560)
Supplement: S1 Table — (PPTX) [file pone.0226560.s004.pptx]

## Slide 1
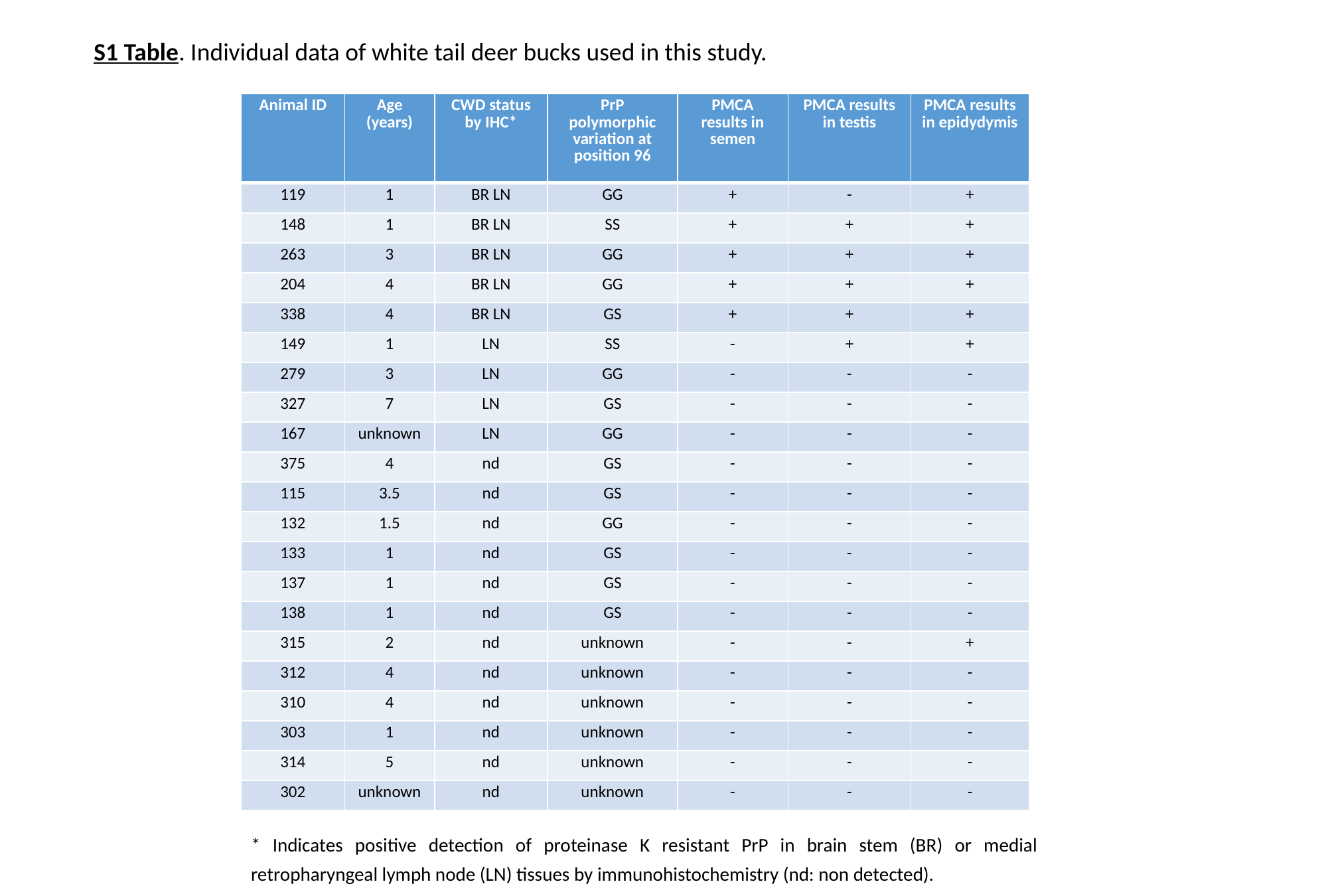

S1 Table. Individual data of white tail deer bucks used in this study.
| Animal ID | Age (years) | CWD status by IHC\* | PrP polymorphic variation at position 96 | PMCA results in semen | PMCA results in testis | PMCA results in epidydymis |
| --- | --- | --- | --- | --- | --- | --- |
| 119 | 1 | BR LN | GG | + | - | + |
| 148 | 1 | BR LN | SS | + | + | + |
| 263 | 3 | BR LN | GG | + | + | + |
| 204 | 4 | BR LN | GG | + | + | + |
| 338 | 4 | BR LN | GS | + | + | + |
| 149 | 1 | LN | SS | - | + | + |
| 279 | 3 | LN | GG | - | - | - |
| 327 | 7 | LN | GS | - | - | - |
| 167 | unknown | LN | GG | - | - | - |
| 375 | 4 | nd | GS | - | - | - |
| 115 | 3.5 | nd | GS | - | - | - |
| 132 | 1.5 | nd | GG | - | - | - |
| 133 | 1 | nd | GS | - | - | - |
| 137 | 1 | nd | GS | - | - | - |
| 138 | 1 | nd | GS | - | - | - |
| 315 | 2 | nd | unknown | - | - | + |
| 312 | 4 | nd | unknown | - | - | - |
| 310 | 4 | nd | unknown | - | - | - |
| 303 | 1 | nd | unknown | - | - | - |
| 314 | 5 | nd | unknown | - | - | - |
| 302 | unknown | nd | unknown | - | - | - |
* Indicates positive detection of proteinase K resistant PrP in brain stem (BR) or medial retropharyngeal lymph node (LN) tissues by immunohistochemistry (nd: non detected).
